# New insights in the discovery of novel *h*-MAO-B inhibitors: structural characterization of a series of *N*-phenyl-4-oxo-4*H*-chromene-3-carboxamide derivatives

**DOI:** 10.1107/S2056989015007859

**Published:** 2015-04-25

**Authors:** Ligia R. Gomes, John Nicolson Low, Fernando Cagide, Daniel Chavarria, Fernanda Borges

**Affiliations:** a FP-ENAS-Faculdade de Ciências de Saúde, Escola Superior de Saúde da UFP, Universidade Fernando Pessoa, Rua Carlos da Maia, 296, P-4200-150 Porto, Portugal; b Department of Chemistry, University of Aberdeen, Meston Walk, Old Aberdeen AB24 3UE, Scotland; c CIQ/Departamento de Quιmica e Bioquιmica, Faculdade de Ciências, Universidade do Porto, 4169-007 Porto, Portugal

**Keywords:** crystal structure, drug design, chromones, conformation, supra­molecular structure, hydrogen bonding

## Abstract

Six *N*-substituted-phenyl 4-oxo-4*H*-chromene-3-carboxamides, namely *N*-(2-nitro­phen­yl)-4-oxo-4*H*-chromene-3-carboxamide, C_16_H_10_N_2_O_5_ (2*b*), *N*-(3-meth­oxy­phen­yl)-4-oxo-4*H*-chromene-3-carboxamide, C_17_H_13_NO_4_, (3*a*), *N*-(3-bromo­phen­yl)-4-oxo-4*H*-chromene-3-carboxamide, C_16_H_10_BrNO_3_, (3*b*), *N*-(4-methoxy­phen­yl)-4-oxo-4*H*-chromene-3-carboxamide, C_17_H_13_NO_4_, (4*a*), *N*-(4-methyl­phen­yl)-4-oxo-4*H*-chromene-3-carboxamide, C_17_H_13_NO_3_, (4*d*), and *N*-(4-hy­droxy­phen­yl)-4-oxo-4*H*-chromene-3-carboxamide, C_16_H_11_NO_4_, (4*e*), have been structurally characterized. All compounds exhibit an *anti* conformation with respect to the C—N rotamer of the amide and a *trans-*related conformation with the carbonyl groups of the chromone ring of the amide. These structures present an intra­molecular hydrogen-bonded network comprising an N—H⋯O hydrogen bond between the amide N atom and the O atom of the carbonyl group of the pyrone ring, forming an *S*(6) ring, and a weak C_ar_—H⋯O hydrogen bond in which the carbonyl group of the amide acts as acceptor for the H atom of an *ortho*-C atom of the exocyclic phenyl ring, which results in another *S*(6) ring. The N—H⋯O intra­molecular hydrogen bond constrains the carboxamide moiety such that it is virtually coplanar with the chromone ring.

## Abstract 2

*N*-(Substituted phen­yl)-4-oxo-4*H*-chromene-3-carboxamides have very similar conformations but show different inhibition activities against *h*-MAO-B so it may be assumed that the electronic environment provided by the substituents on the phenyl ring is the primary condition for the pharmacological activities displayed by these mol­ecules.

## Chemical context   

Chromones are a group of natural and synthetic oxygen heterocyclic compounds having a high degree of chemical diversity that is frequently linked to a broad array of biological activities. The chromone-3-(phen­yl)carboxamide derivatives, depicted the scheme, have emerged as promising compounds for the management of neurodegenerative diseases such as Alzheimer’s and Parkinson’s since they display selective inhibition activities against *h*-MAO-B. Recent data (Cagide *et al.*, 2015 ▸) suggest that the activity and selectivity towards that enzyme is dependent on the nature and position of the substituent located in the exocyclic phenyl ring. When compared with the unsubstituted compound (1), the *para* substitution in the exocyclic phenyl ring seems to play an important role in the enzymatic inter­action: the presence of *para*-Cl (4*c*) and –CH_3_ (4*d*) substituents favours the potency while an –OH (4*e*) substituent has the opposite effect. The data acquired so far point out the importance of a structure–activity relationship study to optimize the potency *vs* selectivity of this type of inhibitor, namely performing structural and electronic changes in the substituents.

Thus, the results for the structural characterization of some chromone-3-phenyl­carboxamide derivatives are presented and discussed. These compounds are as follows – (1): *N*-phenyl-4-oxo-4*H*-chromene-3-carboxamide (Cagide *et al.*, 2015 ▸); (2*a*): *N*-(2-meth­oxy­phen­yl)-4-oxo-4*H*-chromene-3-carb­oxamide (Gomes *et al.*, 2013 ▸); (2*b*): *N*-(2-nitro­phen­yl)-4-oxo-4*H*-chromone-3-carboxamide (CCDC 1025354); (3*a*): *N*-(3-meth­oxy­phen­yl)-4-oxo-4*H*-chromene-3-carboxamide (CCDC 102353); (3*b*): *N*-(3-bromo­phen­yl)-4-oxo-4*H*-chromene-3-carboxamide (CCDC 1025352); (4*a*): *N*-(4-meth­oxyphen­yl)-4-oxo-4*H*-chromene-3-carboxamide (CCDC 1025355); (4*b*): *N*-(4-bromo­phen­yl)-4-oxo-4*H*-chromene-3-carboxamide (Gomes *et al.*, 2015 ▸); (4*c*): *N*-(4-chloro­phen­yl)-4-oxo-4*H*-chromene-3-carboxamide (Gomes *et al.*, 2015 ▸); (4*d*): *N*-(4-methyl­phen­yl)-4-oxo-4*H*-chromene-3-carboxamide; (4*e*): *N*-(4-hy­droxy­phen­yl)-4-oxo-4*H*-chromene-3-carboxamide (CCDC 102524). Compounds with CCDC numbers given were deposited by the current authors, Gomes, Borges and Low, in the Cambridge Structural Database (CSD; Groom & Allen, 2014 ▸).
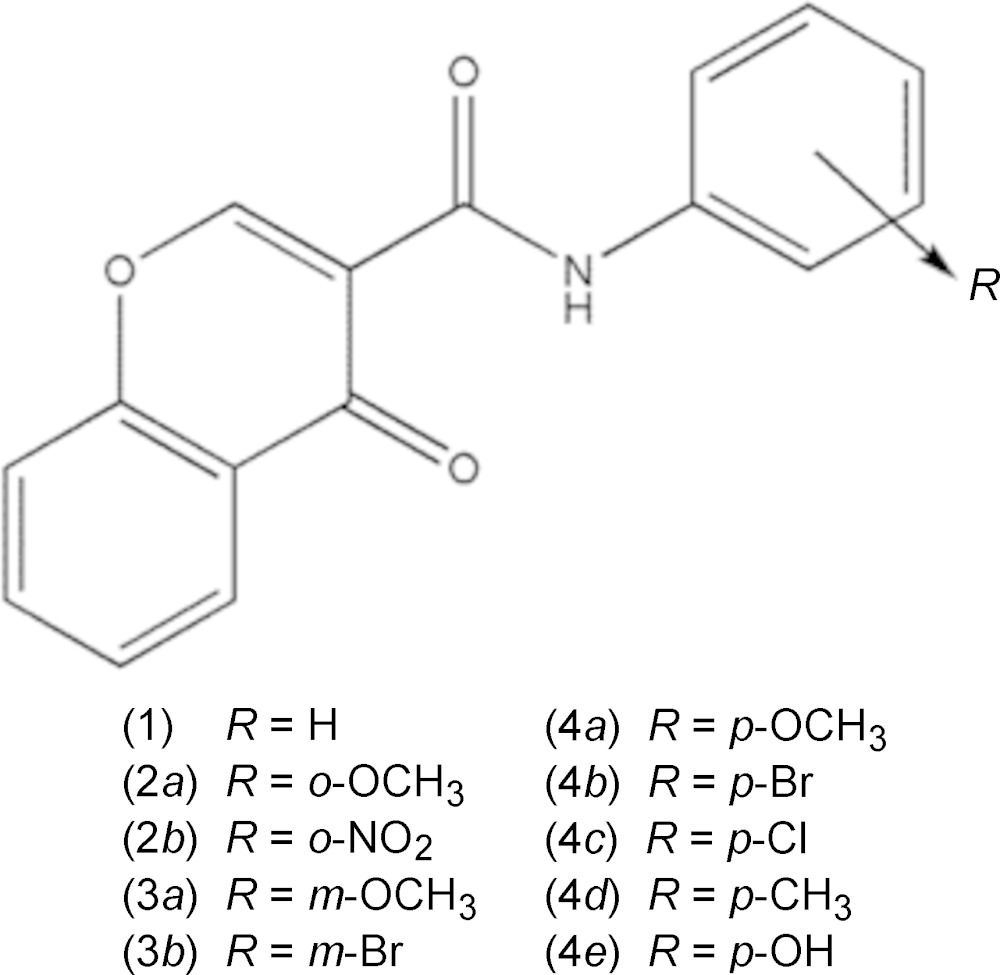


## Structural commentary   

### Mol­ecular structures   

***Conformations and intra­molecular hydrogen-bond network***

**Figure 1:**
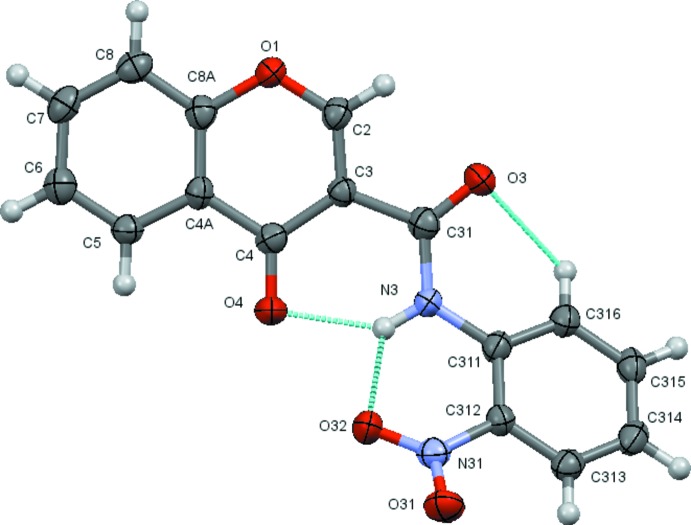
A view of the asymmetric unit of (2*b*) with the atom-numbering scheme. Displacement ellipsoids are drawn at the 70% probability level.

The structural analysis confirms that the mol­ecules are 4-chromone derivatives with a phenyl­amide substituent on position number 3 of the pyrone ring. Fig. 1 ▸ to 6 show the displacement ellipsoid diagrams with the adopted labelling scheme for (2*b*), (3*a*), (3*b*), (4*a*), (4*d*) and (4*e*), the structurally characterized compounds in this work. As seen, the mol­ecules exhibit an *anti* conformation with respect to the C–N rotamer of the amide following a pattern given by compound (1), which was previously described by Cagide *et al.* (2015 ▸). Due to the asymmetry of the chromone residue, the *anti* conformation can assume several geometries depending on the relative position of the carbonyl groups of the chromone ring and the amide group which can be *cis* or *trans* related. Compounds (1)–(4) exhibit a *trans* relation between these bonds as can be seen in Figs. 1 ▸ ▸ ▸ ▸ ▸ ▸ to 6. This mol­ecular conformation allows the establishment of two or three intra­molecular hydrogen bonds. Details of the intra­molecular hydrogen bonding are given in Tables 2 ▸–7 ▸ ▸ ▸ ▸ ▸. Generally, as seen in the scheme below, there is an intra­molecular hydrogen bond involving the amide and the chromone where the amide nitro­gen atom acts as donor to the oxo oxygen atom of the chromone ring, forming an S(6) ring; the carboxyl oxygen of the amide acts as acceptor for a weak H inter­action with the C–H group located at the *ortho* position of the phenyl ring, forming another S(6) ring. This hydrogen-bonding network probably enhances the planarity of the mol­ecules and may prevent them from adopting some other possible conformations by restraining their geometries. Compounds (2*a*) and (2*b*) have substituents located at the *ortho* position on the benzyl ring with oxygen atoms (meth­oxy and nitro, respectively) that act as acceptors for the amide nitro­gen atom of the carboxamide residue, hence forming a third intra­molecular hydrogen bond (see scheme).
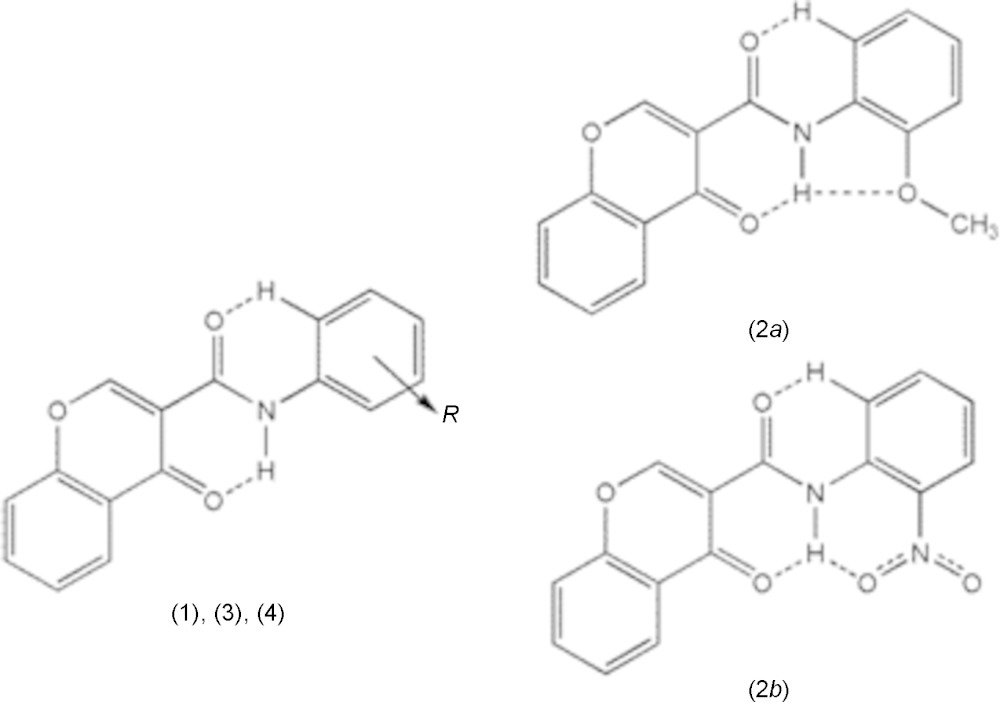


***Mol­ecular geometries***

The values for bond lengths involving the atoms of the carboxamide residue assume the expected ranges for amides with aromatic substituents. The C3—C31 bond ranges from 1.49 to 1.51 Å, which are the typical range values for an C*sp* ^3^—C*sp* ^3^ bond (Allen *et al.*, 1987 ▸). The C31—O3 bond lengths range from 1.22 to 1.25 Å and the C31—N3 bond lengths are within the 1.33 to 1.37 Å inter­val, showing the the partial *sp* ^2^ character of the amide nitro­gen atom attributed to those compounds.

Table 1 ▸ details selected dihedral angles between the mean planes of aromatic rings, θ_Chr-Phe_, between the chromone ring and the amide moiety (the plane defined by atoms O3, C31and N3), θ_Chr-amide_, and between the exocyclic phenyl ring and the amide, θ_Phe-amide_. Those dihedral angles are primarily due to the rotation of the rings around the C3—C31 and N3—C311 bonds with exception of (3*a*) that assumes mainly a bent conformation between the rings. The structural analysis of (1) performed previously (Cagide *et al.*, 2015 ▸) revealed that the amide moiety is practically planar with the chromone ring: it makes a dihedral angle of 4.31 (12)° with the plane defined by the O, C and N atoms of the amide residue. The loss of planarity for the overall mol­ecule results from the slight twist of the exocyclic phenyl substituent around the amidic N—C bond, which is the main factor affecting the value for the dihedral angle of 9.48 (12)° between the best plane of the exocyclic phenyl ring and the O—C—N amidic plane. The dihedral angle between the mean plane of the chromone ring and that of the exocyclic phenyl ring is 10.77 (4)°. The θ_Chr-amide_ dihedral angles for the substituted compounds are below 15° for all the compounds, suggesting that the amide moiety is essentially planar with the chromone ring. The strong N3—H3⋯O4 hydrogen contact may preclude higher rotations around the C3—C31 bond in spite of its C*sp* ^3^—C*sp* ^3^ character. The θ_Phe-amide_ angles present more widely spread values, ranging between 2 and 33°. The substituents with oxygen atoms located at the *ortho* position on the exocyclic phenyl ring in (2) which, simultaneously, cause steric hindrance and act as acceptors for the hydrogen atom of the amide, thus forming an intra­molecular hydrogen bond, suggest that a tricky balance between those two factors allows the formation of several energetically accessible rotated conformations. This fact is especially noticeable in the various conformation polymorphs of (2*a*).

The remaining compounds are not constrained by steric hindrance of the *ortho*-substituents but they still present a wide range of values for the θ_Phe-amide_ dihedral angles (between 3 and 24°). The θ_Chr-Phe_ values may be used as a measure of the relative positioning of the two aromatic rings which may define the primary conformation for the mol­ecules. The aromatic rings are usually rotated or co-planar, with exception of (3*a*) where they are bent with respect to each other. The chromones with halogen substituents assume the most planar conformations, probably related to the typical positive mesomeric effects on the π system. Considering the fact that the *para*-substituent on the exocyclic phenyl ring for chromone-3-phenyl­carboxamides has a positive effect on their activity, and the requirement of establishing the factors that can modulate the enzyme–ligand inter­action, it can be assumed their *h*-MAO-B activity is strongly dependent on the electronic environment of the substituent. This is not a preferred conformation that reduces or enhances the activity, so it may be assumed that the electronic environment provided by the substituent is the primary condition for the pharmacological activities displayed by those mol­ecules.

In compound (3*b*) there are two mol­ecules in the asymmetric unit. A calculation using *Molfit* with Quaternion Transformation Method (Mackay, 1984 ▸) gave the following fit: weighted/unit weight r.m.s. fits: 0.133/0.144 Å for 23 atoms with mol­ecule 1 inverted on mol­ecule 2, 21 atoms. The largest individual displacement is 0.178 Å(Br13/Br23). The r.m.s. bond fit = 0.0052 Å and the r.m.s. angle fit = 0.437°.

## Supra­molecular features   

The carboxamide H atom is not involved in any inter­molecular inter­action in any of the compounds.

**Figure 7:**
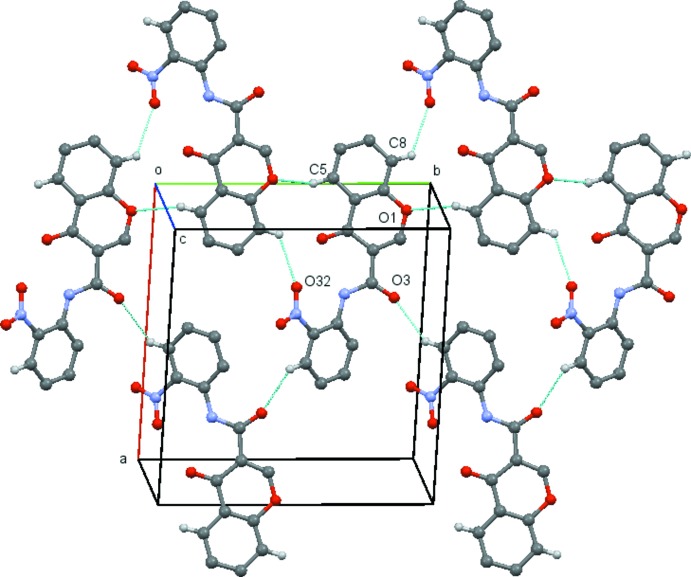
View of the sheet formed by the inter­connection of three C—H⋯O hydrogen bonded chains in compound (2*b*). Hydrogen atoms not involved in the hydrogen bonding have been omitted for clarity. [Symmetry codes (from bottom to top rows and left to right). Bottom: −*x* + 1, *y* − 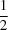, −*z* + 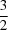; −*x* + 1, *y* + 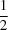, −*z* + 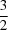. Middle: *x*, −*y*, *z*; *x*, *y*, *z*; *x*, *y* + 1, *z*. Top: −*x*, *y* − 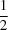, −*z* + 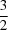; −*x*, *y* + 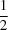, −*z* + 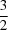.]

In (2*b*), the mol­ecules are linked by C8—H8⋯O32(−*x*, *y* + 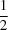, −*z* + 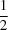), C5—H5⋯O1(−*x*, *y* − 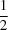, −*z* + 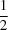) and C313—H313⋯O3(−*x*, *y* − 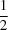, −*z* + 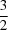) hydrogen bonds which, by the action of twofold screw axes running parallel to the *b axis*, link the mol­ecules into corrugated sheets which lie parallel to the (10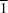) plane, and which form a distorted chequerboard pattern comprised of 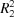
(15) and 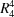
(23) rings (Table 2 ▸ and Fig. 7 ▸).

**Figure 8:**
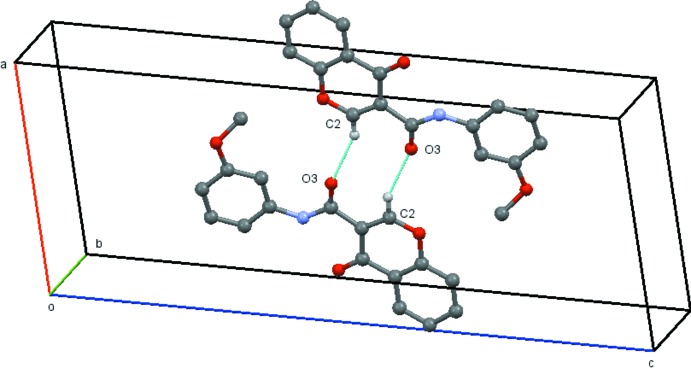
View of the dimer formed across the inversion centre (½, ½, ½) in (3*a*). Hydrogen atoms not involved in the hydrogen bonding have been omitted for clarity.

In (3*a*), the mol­ecules are linked by the C2—H2⋯O3(−*x* + 1, −*y* + 1, −*z* + 1) hydrogen bond, forming centrosymmetric dimers across the inversion centre at (1/2, 1/2, 1/2) (Table 3 ▸ and Fig. 8 ▸).

**Figure 9:**
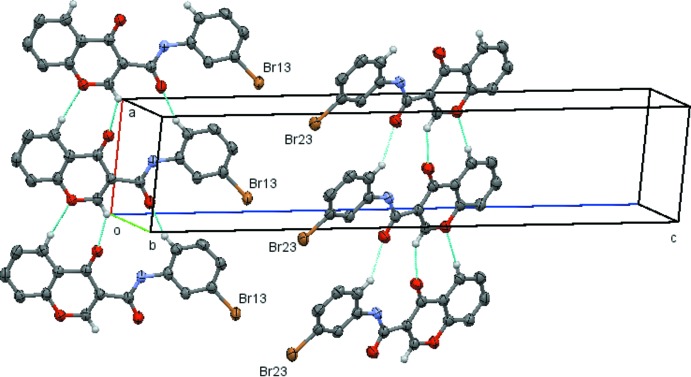
View of the two independent ladders formed linked 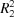
(13) rings which run parallel to the *a* axis in compound (3*b*). Hydrogen atoms not involved in the hydrogen bonding have been omitted for clarity. [Symmetry codes (bottom to top): *x* − 1, *y*, *z*; *x*, *y*, *z*; *x* + 1, *y*, *z*.]

In (3*b*), independent ladders of mol­ecule 1 and mol­ecule 2 are propagated along the *a*-axis direction by unit translation. These are formed by chains of 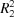
(13) rings produced by the weak C*x*2—H*x*2⋯O*x*4(*x* + 1, *y*, *z*) and C*x*36—H*x*36⋯O*x*3(*x* − 1, *y*, *z*) inter­actions, where *x* = 1 or 2 (Table 4 ▸ and Fig. 9 ▸).

**Figure 10:**
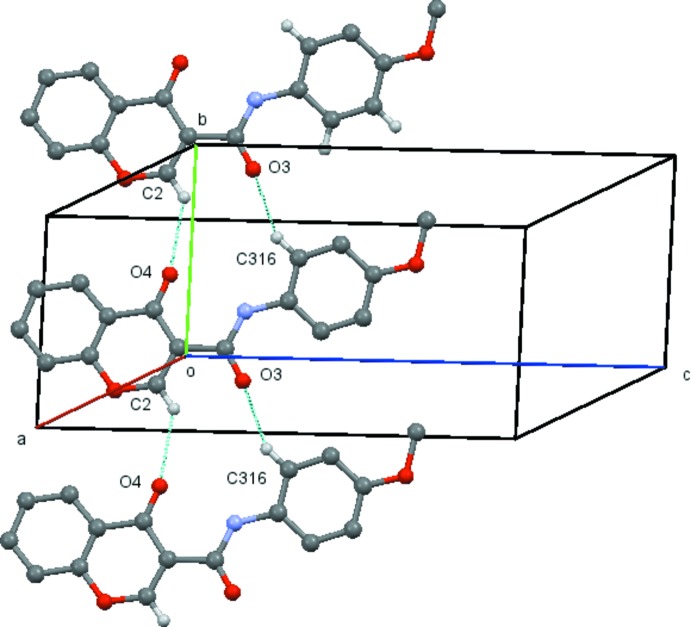
View of the ladder formed by the linked 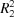
(13) rings which run parallel to the *b* axis in compound (4*a*). Hydrogen atoms not involved in the hydrogen bonding have been omitted for clarity. [Symmetry codes (bottom to top): *x*, *y* − 1, *z*; *x*, *y*, *z*; *x*, *y* + 1, *z.*]

**Figure 11:**
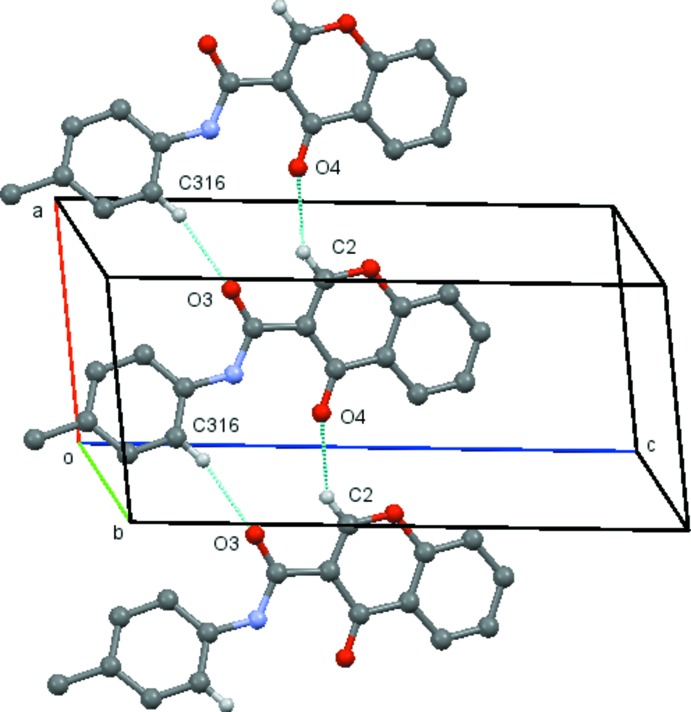
View of the ladder formed by the linked 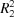
(13) rings which run parallel to the *a* axis in compound (4*d*). Hydrogen atoms not involved in the hydrogen bonding have been omitted for clarity. [Symmetry codes (bottom to top): *x* − 1, *y*, *z*; *x*, *y*, *z*; *x* + 1, *y*, *z*.]

A common feature found for compounds with *para* substituents, (4*a*)–(4*d*) is the formation of a ladder structure composed of mol­ecules propagated by unit axial translations involving inter­molecular hydrogen bonds between C2 and O4 of the chromone ring and the C atom located at the *ortho* position of the exocyclic phenyl ring and the carboxamide O atom. This is also found in (1) and in compound (3*b*), which has a Br substituent located at the *meta* position, in which the ladder structure is supplemented by an inter­molecular hydrogen bond between C5 and O1 of the chromone moiety. In (4*a*), the mol­ecules are linked by C2—H2⋯O4 (*x*, *y* − 1, *z*) and C316—H316⋯O3 (*x*, *y* + 1, *z*) hydrogen bonds, forming 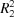
(13) rings structures which are propagated along the *b*-axis direction by unit translation (Table 5 ▸ and Fig. 10 ▸). In (4*d*), the mol­ecules are linked by C2—H2⋯O4(*x* + 1, *y*, *z*) and C316—H316⋯O3(*x* − 1, *y*, *z*) hydrogen bonds, forming 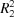
(13) ring structures which are propagated along the *a*-axis direction by unit translation (Table 6 ▸ and Fig. 11 ▸).

**Figure 12:**
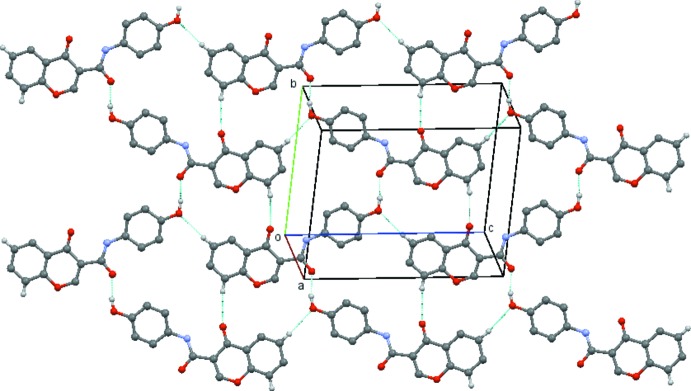
View of the sheet formed by the inter­connection of three C—H⋯O hydrogen-bonded chains in compound (4*e*). Hydrogen atoms not involved in the hydrogen bonding have been omitted for clarity. [Symmetry codes (from bottom to top rows and left to right). Bottom: *x* + 1, *y* − 1, *z* − 1; *x* + 1, *y* − 1, *z* − 1; *x* + 1, *y* − 1, *z* + 1. Middle two rows: *x*, *y*, *z* − 1; *x*, *y*, *z*; *x*, *y*, *z* + 1. Top: *x* − 1, *y* + 1, *z* − 1; *x* − 1, *y* + 1,*z*; *x* − 1, *y* + 1, *z* + 1.]

In the hydroxyl compound (4*e*), the mol­ecules in the asymmetric unit are linked by the O114—H114⋯O23 hydrogen bond, forming a dimer. These dimers are linked by the O214—H214⋯O13(*x* − 1,1 + *y*, *z*) and weak C16—H16⋯O114(*x*, *y*, *z* − 1), C18—H18⋯O24(*x* + 1, *y* − 1, *z* − 1), C26—H26⋯O214(*x*, *y*, *z* + 1) and C28—H28⋯O14(*x*, *y*, *z* + 1) hydrogen bonds, which link the mol­ecules into sheets that form a chequerboard pattern and which lie parallel to the (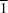
10) plane, comprised of 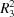
(15) and 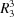
(24) rings (Table 7 ▸ and Fig. 12 ▸).

Selected π–π contacts, with centroid-to-centroid distances less than 4.0 Å and with angles between planes of less than 10° for compounds (2*b*), (3*b*), (4*a*) and (4*d*) are listed in Table 8 ▸. No inter­actions were found for (3*a*).

## Synthesis and crystallization   

The compounds were obtained by synthetic strategies described elsewhere (Cagide *et al.*, 2011 ▸). Chromone-3-carboxamide derivatives were synthesized using chromone-3-carb­oxy­lic acid as starting material which, after *in situ* activ­ation with phospho­rus(V) oxychloride (POCl_3_) in di­methyl­formamide, react with the different substituted anilines. Crystals were recrystallized from ethyl­acetate forming colourless plates whose dimensions are given in Table 9 ▸.

## Refinement   

Crystal data, data collection and structure refinement details are summarized in Table 9 ▸.

In (3*b*) there are two mol­ecules in the asymmetric unit. The largest difference map peaks are associated with the Br atoms.

In all compounds, H atoms attached to C atoms were treated as riding atoms with C—H(aromatic) = 0.95 Å with *U*_iso_(H) = 1.2*U*_eq_(C); C—H(meth­yl), = 0.98 Å with *U*_iso_= 1.5*U*_eq_(C). In all compounds, the amino H atoms were refined with the exception of (3*b*) where these atoms were refined as riding atoms with N—H = 0.88 Å with *U*_iso_ = 1.2*U*_eq_(C) and in (4*e*) in which the positional parameters of the amino and hydroxyl H atoms were refined but their *U*_iso_ values were constrained to be *U*_iso_(N) = 1.2*U*_eq_(N) and *U*_iso_(O)b= 1.5*U*_eq_(O). The final positions of these atoms were checked in a difference Fourier map, as were the positions of the H atoms in any methyl groups. The quality of the crystals for (4*e*) was poor and the crystals were twinned. The completeness is 97%. The crystal studied was refined as a two-component twin [twin law: 2-axis (001) [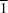
05], BASF = 0.40].

## Supplementary Material

Crystal structure: contains datablock(s) 2b, 3a, 3b, 4a, 4d, 4e, global. DOI: 10.1107/S2056989015007859/lh5762sup1.cif

Structure factors: contains datablock(s) 2b. DOI: 10.1107/S2056989015007859/lh57622bsup2.hkl

Structure factors: contains datablock(s) 3a. DOI: 10.1107/S2056989015007859/lh57623asup3.hkl

Structure factors: contains datablock(s) 3b. DOI: 10.1107/S2056989015007859/lh57623bsup4.hkl

Structure factors: contains datablock(s) 4a. DOI: 10.1107/S2056989015007859/lh57624asup5.hkl

Structure factors: contains datablock(s) 4d. DOI: 10.1107/S2056989015007859/lh57624dsup6.hkl

Structure factors: contains datablock(s) 4e. DOI: 10.1107/S2056989015007859/lh57624esup7.hkl

Supporting information file. DOI: 10.1107/S2056989015007859/lh57622bsup8.cml

Supporting information file. DOI: 10.1107/S2056989015007859/lh57623asup9.cml

Supporting information file. DOI: 10.1107/S2056989015007859/lh57623bsup10.cml

Supporting information file. DOI: 10.1107/S2056989015007859/lh57624asup11.cml

Supporting information file. DOI: 10.1107/S2056989015007859/lh57624dsup12.cml

Supporting information file. DOI: 10.1107/S2056989015007859/lh57624esup13.cml

CCDC references: 1025354, 1025353, 1025352, 1025255, 1025257, 1025254

Additional supporting information:  crystallographic information; 3D view; checkCIF report

## Figures and Tables

**Figure 2:**
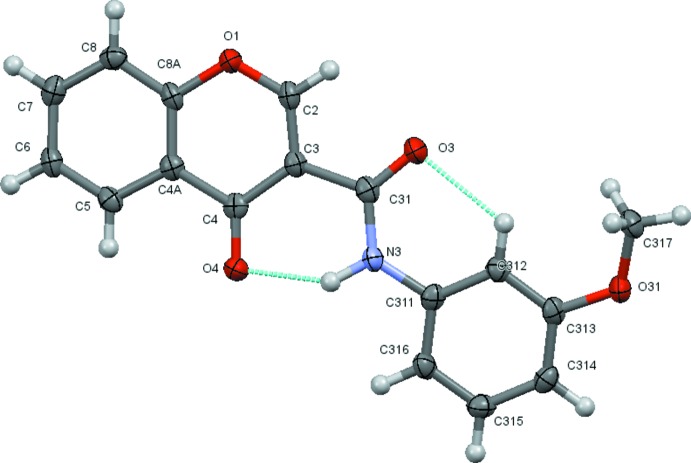
A view of the asymmetric unit of (3*a*) with the atom-numbering scheme. Displacement ellipsoids are drawn at the 70% probability level.

**Figure 3:**
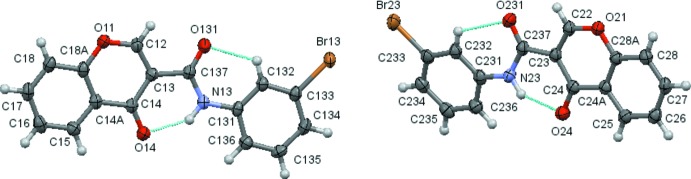
A view of the asymmetric unit of (3*b*) with the atom-numbering scheme. Displacement ellipsoids are drawn at the 70% probability level.

**Figure 4:**
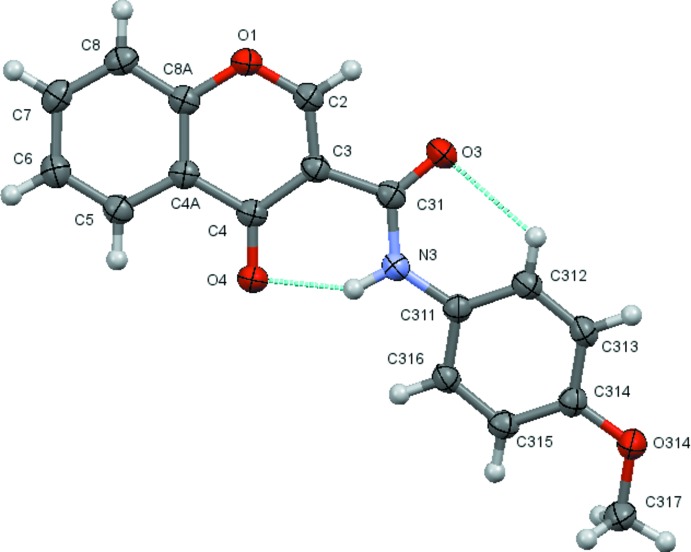
A view of the asymmetric unit of (4*a*) with the atom-numbering scheme. Displacement ellipsoids are drawn at the 70% probability level.

**Figure 5:**
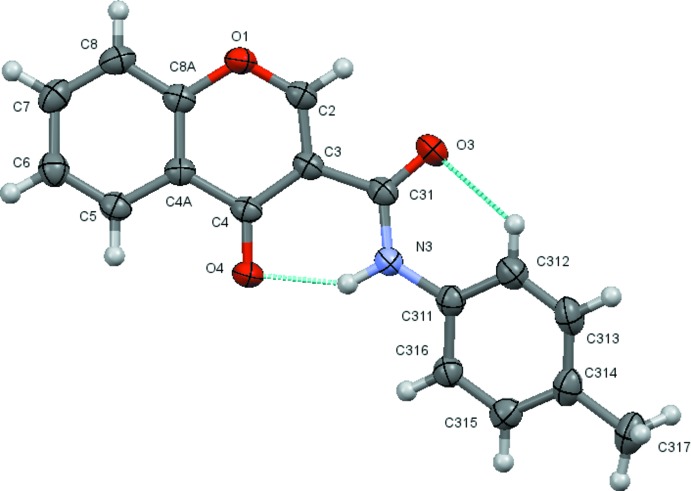
A view of the asymmetric unit of (4*d*) with the atom-numbering scheme. Displacement ellipsoids are drawn at the 70% probability level.

**Figure 6:**
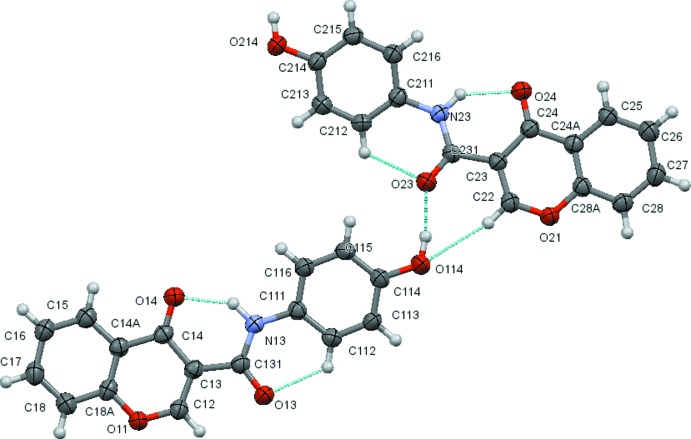
A view of the asymmetric unit of (4*e*) with the atom-numbering scheme. Displacement ellipsoids are drawn at the 70% probability level.

**Table 1:** Selected dihedral angles (°) θ_Chr-Phe_ is the dihedral angle between the mean planes of the chromene and the phenyl ring. θ_Chr-amide_ is the dihedral angle between the mean planes of the chromone ring and the plane defined by atoms O3, C31 and N3. θ_amide-Phe_ is the dihedral angle between the mean planes of the phenyl ring and the plane defined by atoms O3, C31 and N3. The suffices A and B for compound (2*a*) denote the polymeric forms. Basic Conf. denotes the primary shape given by the relative position of the aromatic rings around the carboxamide linkage.

Compound	θ_Chr-Phe_	θ_Chr-amide_	θ_amide-Phe_	Basic Conf.
(1)	10.77 (4)	4.31 (12)	9.48 (12)	Rotation
(2*a* mol1_A_	11.64 (5)	8.72 (14)	20.35 (13)	Rotation
(2*a* mol2_A_	2.47 (5)	1.75 (2)	2.2 (2)	Planar
(2*a* mol1_B_	6.50 (18)	15.0 (5)	10.1 (6)	Rotation
(2*a* mol2_B_	10.52 (17)	1.8 (6)	12.27 (6)	Rotation
(2*b*)	35.96 (9)	2.35 (4)	33.6 (2)	Rotation
(3*a*)	15.61 (8)	9.3 (3)	11.7 (2)	Bent
(3*b*) mol1	2.68 (10)	2.0 (4)	4.0 (4)	Planar
(3*b*) mol2	10.31 (12)	0.6 (4)	10.42 (12)	Rotation
(4*a*)	11.48 (6)	5.2 (5)	6.5 (4)	Rotation
(4*b*)	4.90 (10)	2.0 (4)	2.9 (4)	Planar
(4*c*)	1.95 (7)	5.7 (3)	4.4 (3)	Planar
(4*d*)	22.88 (4)	2.71 (8)	23.90 (5)	Rotation
(4*4e*) mol1	3.58 (17)	5.9 (2)	9.5 (3)	Rotation
(4*4e*) mol2	10.02 (15)	10.69 (2)	19.8 (2)	Rotation

**Table 2:** Hydrogen-bond geometry (Å, °) for (2*b*)

*D*—H⋯*A*	*D*—H	H⋯*A*	*D*⋯*A*	*D*—H⋯*A*
N3—H3⋯O4	0.96 (4)	1.95 (4)	2.718 (3)	136 (3)
N3—H3⋯O32	0.96 (4)	1.96 (4)	2.633 (3)	126 (3)
C316—H316⋯O3	0.95	2.40	2.902 (4)	113
C8—H8⋯O32^i^	0.95	2.58	3.210 (4)	124
C5—H5⋯O1^ii^	0.95	2.60	3.375 (4)	139
C313—H313⋯O3^iii^	0.95	2.49	3.299 (4)	143

Symmetry codes: (i) 
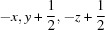
; (ii) 
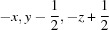
; (iii) 
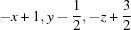
.

**Table 3:** Hydrogen-bond geometry (Å, °) for (3*a*)

*D*—H⋯*A*	*D*—H	H⋯*A*	*D*⋯*A*	*D*—H⋯*A*
N3—H3⋯O4	0.95 (2)	1.89 (2)	2.7147 (17)	143.8 (18)
C312—H312⋯O3	0.95	2.25	2.855 (2)	121
C2—H2⋯O3^i^	0.95	2.37	3.243 (2)	153

Symmetry code: (i) 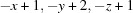.

**Table 4:** Hydrogen-bond geometry (Å, °) for (3*b*)

*D*—H⋯*A*	*D*—H	H⋯*A*	*D*⋯*A*	*D*—H⋯*A*
N13—H13⋯O14	0.88	1.93	2.686 (3)	143
N23—H23⋯O24	0.88	1.94	2.698 (3)	143
C12—H12⋯O131	0.95	2.34	2.727 (4)	104
C22—H22⋯O231	0.95	2.33	2.725 (4)	104
C132—H132⋯O131	0.95	2.26	2.860 (4)	121
C232—H232⋯O231	0.95	2.28	2.865 (4)	119
C12—H12⋯O14^i^	0.95	2.49	3.221 (4)	134
C22—H22⋯O24^i^	0.95	2.43	3.185 (4)	136
C15—H15⋯O11^ii^	0.95	2.68	3.587 (4)	160
C25—H25⋯O21^ii^	0.95	2.58	3.530 (4)	177
C136—H136⋯O131^ii^	0.95	2.43	3.282 (4)	149
C236—H236⋯O231^ii^	0.95	2.41	3.270 (4)	151

Symmetry codes: (i) 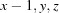; (ii) 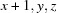.

**Table 5:** Hydrogen-bond geometry (Å, °) for (4*a*)

*D*—H⋯*A*	*D*—H	H⋯*A*	*D*⋯*A*	*D*—H⋯*A*
N3—H3⋯O4	0.901 (17)	1.903 (16)	2.6919 (13)	145.0 (15)
C312—H312⋯O3	0.95	2.37	2.9441 (17)	119
C2—H2⋯O4^i^	0.95	2.47	3.212 (3)	134
C316—H316⋯O3^ii^	0.95	2.33	3.201 (2)	152

Symmetry codes: (i) 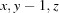; (ii) 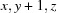.

**Table 6:** Hydrogen-bond geometry (Å, °) for (4*d*)

*D*—H⋯*A*	*D*—H	H⋯*A*	*D*⋯*A*	*D*—H⋯*A*
N3—H3⋯O4	0.900 (18)	1.916 (18)	2.7098 (13)	146.1 (15)
C312—H312⋯O3	0.95	2.37	2.9240 (16)	116
C2—H2⋯O4^i^	0.95	2.40	3.1280 (14)	133
C316—H316⋯O3^ii^	0.95	2.44	3.3644 (14)	164

Symmetry codes: (i) 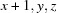; (ii) 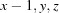.

**Table 7:** Hydrogen-bond geometry (Å, °) for (4*e*)

*D*—H⋯*A*	*D*—H	H⋯*A*	*D*⋯*A*	*D*—H⋯*A*
N13—H13⋯O14	0.94 (4)	1.88 (4)	2.693 (4)	143 (4)
N23—H23⋯O24	0.90 (4)	1.95 (4)	2.698 (4)	139 (4)
C112—H112⋯O13	0.95	2.23	2.833 (4)	121
C212—H212⋯O23	0.95	2.28	2.845 (4)	117
O114—H114⋯O23	0.91 (6)	1.76 (6)	2.647 (4)	167 (5)
O214—H214⋯O13^i^	0.88 (5)	1.81 (5)	2.668 (4)	165 (5)
C16—H16⋯O114^ii^	0.95	2.46	3.411 (5)	174
C18—H18⋯O24^iii^	0.95	2.56	3.481 (5)	163
C22—H22⋯O114	0.95	2.58	3.508 (4)	166
C26—H26⋯O214^iv^	0.95	2.51	3.454 (5)	175
C28—H28⋯O14^iv^	0.95	2.46	3.391 (5)	165

Symmetry codes: (i) 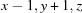; (ii) 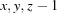; (iii) 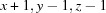; (iv) 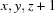.

**Table 8:** Selected π–π contacts (Å, °) for compounds (2*b*), (3*b*) (mol­ecule 1), (4*a*) and (4*d*) *Cg*1, *Cg*2 and *Cg*3(*Cg*7) are the centroids of the pyrone, of the chromone phenyl and of the carboxamide phenyl rings, respectively. * indicates contacts in which the planes involved are inclined to each other, the perpendicular distance between the planes is an average value and the angle between the planes is given in place of a slippage. Only inter­planar inter­actions with *Cg*⋯*Cg* distances less than or equal to 4.0 Å or with angles between the planes of less than 10° are included.

Compound	contacts	distance	perp. distance	Slippage^*^
(2*b*)	*Cg*1⋯*Cg*1^iii^	3.859 (3)	3.4223^*^	4.0 (13)^*^
	*Cg*1⋯*Cg*2^iv^	3.564 (3)	3.3951^*^	3.86 (13)^*^
	*Cg*2⋯*Cg*2^iv^	3.674 (3)	3.4035^*^	4.0 (13)^*^
	*Cg*3⋯*Cg*3^i^	3.649 (3)	3.3049 (11)	1.546
(3*b*)	*Cg*1⋯*Cg*3^v^	3.6621 (17)	3.4150^*^	2.91 (13)
	*Cg*2⋯*Cg*3^vi^	3.6851 (18)	3.3587^*^	2.47 (14)^*^
	*Cg*2⋯*Cg*3^v^	3.7278 (17)	3.4360^*^	2.47 (14)^*^
(4*a*)	*Cg*2⋯*Cg*3^ii^	3.780 (3)	3.383^*^	1.90 (6)^*^
(4*d*)	*Cg*1⋯*Cg*1^vii^	3.4831 (7)	3.3257 (4)	1.035
	*Cg*1⋯*Cg*2^Vii^	3.6037 (7)	3.3137^*^	2.46 (5)^*^
(4*e*)	*Cg*1⋯*Cg*3^vi^	3.669 (2)	3.3741^*^	3.50 (17)^*^
	*Cg*1⋯*Cg*7^v^	3.768 (2)	3.3792^*^	3.09 (17)^*^

Symmetry codes: (i) 1 − *x*, 1 − *y*, 1 − *z*; (ii) 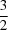 − *x*, −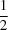 + *y*, 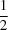 − *z*; (iii) *x*, 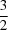 − *y*, −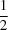 + *z*; (iv) *x*, 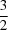 − *y*, 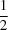 + *z*; (v) 1 − *x*, 1 − *y*, −*z*; (vi) 1 − *x*, −*y*, −*z*; (vii) 1 − *x*, −*y*, 1 − *z*.

**Table:** 

	(2*b*)	(3*a*)	(3*b*)
Crystal data			
Chemical formula	C_16_H_10_N_2_O_5_	C_17_H_13_NO_4_	C_16_H_10_BrNO_3_
*M*_r_	310.26	295.28	344.16
Crystal system, space group	Monoclinic, *P*2_1_/*c*	Monoclinic, *P*2_1_/*n*	Triclinic, *P* 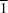
Temperature (K)	100	100	120
*a*, *b*, *c* (Å)	14.104 (9), 12.692 (8), 7.340 (5)	9.6903 (2), 5.5303 (4), 24.9335 (18)	6.7435 (1), 7.3012 (1), 28.0740 (9)
α, β, γ (°)	90, 100.065 (13), 90	90, 99.162 (5), 90	85.309 (4), 89.164 (4), 70.645 (3)
*V* (Å^3^)	1293.7 (15)	1319.15 (14)	1299.64 (5)
*Z*	4	4	4
Radiation type	Mo *K*α	Mo *K*α	Mo *K*α
μ (mm^−1^)	0.12	0.11	3.17
Crystal size (mm)	0.09 × 0.02 × 0.01	0.16 × 0.11 × 0.02	0.38 × 0.34 × 0.06

Data collection			
Diffractometer	Rigaku Saturn724+	Rigaku Saturn724+	Rigaku R-AXIS conversion
Absorption correction	Multi-scan (*CrystalClear-SM Expert*; Rigaku, 2012)	Multi-scan (*CrystalClear-SM Expert*; Rigaku, 2012)	Multi-scan (*CrystalClear-SM Expert*; Rigaku, 2012)
*T*_min_, *T*_max_	0.989, 0.999	0.983, 0.998	0.379, 0.833
No. of measured, independent and observed [*I* > 2σ(*I*)] reflections	8466, 2947, 2215	7859, 2665, 1952	16781, 5939, 5633
*R*_int_	0.061	0.055	0.045
(sin θ/λ)_max_ (Å^−1^)	0.649	0.625	0.650

Refinement			
*R*[*F* ^2^ > 2σ(*F* ^2^)], *wR*(*F* ^2^), *S*	0.077, 0.153, 1.16	0.041, 0.108, 0.98	0.044, 0.116, 1.08
No. of reflections	2947	2665	5939
No. of parameters	212	205	379
H-atom treatment	H atoms treated by a mixture of independent and constrained refinement	H atoms treated by a mixture of independent and constrained refinement	H-atom parameters constrained
Δρ_max_, Δρ_min_ (e Å^−3^)	0.24, −0.31	0.27, −0.28	1.79, −0.86

**Table:** 

	(4*a*)	(4*d*)	(4*e*)
Crystal data			
Chemical formula	C_17_H_13_NO_4_	C_17_H_13_NO_3_	C_16_H_11_NO_4_
*M*_r_	295.28	279.28	281.26
Crystal system, space group	Monoclinic, *P*2_1_/*n*	Triclinic, *P* 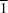	Triclinic, *P* 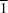
Temperature (K)	100	100	100
*a*, *b*, *c* (Å)	14.1629 (10), 6.772 (5), 15.1898 (11)	6.6106 (5), 7.0143 (5), 15.3749 (11)	7.0756 (5), 12.5125 (9), 14.2944 (10)
α, β, γ (°)	90, 116.607 (11), 90	91.444 (6), 95.238 (6), 112.551 (8)	86.267 (8), 83.839 (8), 84.588 (8)
*V* (Å^3^)	1302.6 (10)	654.25 (9)	1250.68 (16)
*Z*	4	2	4
Radiation type	Mo *K*α	Mo *K*α	Mo *K*α
μ (mm^−1^)	0.11	0.10	0.11
Crystal size (mm)	0.15 × 0.07 × 0.01	0.16 × 0.09 × 0.02	0.14 × 0.04 × 0.04

Data collection			
Diffractometer	Rigaku Saturn724+	Rigaku Saturn724+	Rigaku Saturn724+
Absorption correction	Multi-scan (*CrystalClear-SM Expert*; Rigaku, 2012)	Multi-scan (*CrystalClear-SM Expert*; Rigaku, 2012)	Multi-scan (*CrystalClear-SM Expert*; Rigaku, 2012)
*T*_min_, *T*_max_	0.984, 0.999	0.985, 0.998	0.985, 0.996
No. of measured, independent and observed [*I* > 2σ(*I*)] reflections	16554, 2987, 2617	9400, 2986, 2645	5627, 5627, 4343
*R*_int_	0.042	0.035	
(sin θ/λ)_max_ (Å^−1^)	0.650	0.651	0.652

Refinement			
*R*[*F* ^2^ > 2σ(*F* ^2^)], *wR*(*F* ^2^), *S*	0.037, 0.103, 0.92	0.043, 0.123, 1.08	0.085, 0.252, 1.18
No. of reflections	2987	2986	5627
No. of parameters	204	196	392
H-atom treatment	H atoms treated by a mixture of independent and constrained refinement	H atoms treated by a mixture of independent and constrained refinement	H atoms treated by a mixture of independent and constrained refinement
Δρ_max_, Δρ_min_ (e Å^−3^)	0.39, −0.18	0.33, −0.26	0.41, −0.38

Computer programs: *CrystalClear-SM Expert* (Rigaku, 2012 ▸), *SHELXS97* (Sheldrick, 2008), *SHELXL2014* (Sheldrick, 2015 ▸), *PLATON* (Spek, 2009 ▸), *Flipper 25* (Oszlányi & Sütő, 2004 ▸), *OSCAIL* (McArdle *et al.*, 2004 ▸), *ShelXle* (Hübschle *et al.*, 2011 ▸) and *Mercury* (Macrae *et al.*, 2006 ▸).

## supplementary crystallographic information

### (2b) N-(2-Nitrophenyl)-4-oxo-4H-chromene-3-carboxamide Crystal data

**Table:**

C_16_H_10_N_2_O_5_	*F*(000) = 640
*M**_r_* = 310.26	*D*_x_ = 1.593 Mg m^−^^3^
Monoclinic, *P*2_1_/*c*	Mo *K*α radiation, λ = 0.71075 Å
*a* = 14.104 (9) Å	Cell parameters from 3262 reflections
*b* = 12.692 (8) Å	θ = 2.2–31.3°
*c* = 7.340 (5) Å	µ = 0.12 mm^−^^1^
β = 100.065 (13)°	*T* = 100 K
*V* = 1293.7 (15) Å^3^	Rod, yellow
*Z* = 4	0.09 × 0.02 × 0.01 mm

### (2b) N-(2-Nitrophenyl)-4-oxo-4H-chromene-3-carboxamide Data collection

**Table:**

Rigaku Saturn724+ (2x2 bin mode) diffractometer	2947 independent reflections
Radiation source: Rotating Anode	2215 reflections with *I* > 2σ(*I*)
Confocal monochromator	*R*_int_ = 0.061
Detector resolution: 28.5714 pixels mm^-1^	θ_max_ = 27.5°, θ_min_ = 2.9°
profile data from ω–scans	*h* = −18→18
Absorption correction: multi-scan (*CrystalClear-SM Expert*; Rigaku, 2012)	*k* = −16→15
*T*_min_ = 0.989, *T*_max_ = 0.999	*l* = −9→9
8466 measured reflections	

### (2b) N-(2-Nitrophenyl)-4-oxo-4H-chromene-3-carboxamide Refinement

**Table:**

Refinement on *F*^2^	0 restraints
Least-squares matrix: full	Hydrogen site location: mixed
*R*[*F*^2^ > 2σ(*F*^2^)] = 0.077	H atoms treated by a mixture of independent and constrained refinement
*wR*(*F*^2^) = 0.153	*w* = 1/[σ^2^(*F*_o_^2^) + (0.0365*P*)^2^ + 1.6526*P*] where *P* = (*F*_o_^2^ + 2*F*_c_^2^)/3
*S* = 1.16	(Δ/σ)_max_ < 0.001
2947 reflections	Δρ_max_ = 0.24 e Å^−^^3^
212 parameters	Δρ_min_ = −0.31 e Å^−^^3^

### (2b) N-(2-Nitrophenyl)-4-oxo-4H-chromene-3-carboxamide Special details

**Table:**

Geometry. All e.s.d.'s (except the e.s.d. in the dihedral angle between two l.s. planes) are estimated using the full covariance matrix. The cell e.s.d.'s are taken into account individually in the estimation of e.s.d.'s in distances, angles and torsion angles; correlations between e.s.d.'s in cell parameters are only used when they are defined by crystal symmetry. An approximate (isotropic) treatment of cell e.s.d.'s is used for estimating e.s.d.'s involving l.s. planes.

### (2b) N-(2-Nitrophenyl)-4-oxo-4H-chromene-3-carboxamide Fractional atomic coordinates and isotropic or equivalent isotropic displacement parameters (Å^2^)

**Table:**

	*x*	*y*	*z*	*U*_iso_*/*U*_eq_	
O1	0.06264 (13)	0.90167 (15)	0.2131 (3)	0.0216 (5)	
O3	0.33472 (14)	0.85362 (16)	0.4843 (3)	0.0253 (5)	
O4	0.15266 (14)	0.59520 (16)	0.2912 (3)	0.0261 (5)	
O31	0.37222 (16)	0.41995 (16)	0.8305 (3)	0.0296 (5)	
O32	0.27802 (14)	0.48771 (16)	0.5956 (3)	0.0261 (5)	
N3	0.32255 (17)	0.67310 (19)	0.4733 (3)	0.0200 (5)	
H3	0.278 (3)	0.616 (3)	0.447 (5)	0.049 (11)*	
N31	0.35685 (17)	0.48317 (19)	0.7022 (3)	0.0219 (6)	
C2	0.1510 (2)	0.8769 (2)	0.3047 (4)	0.0209 (6)	
H2	0.1918	0.9337	0.3523	0.025*	
C3	0.18677 (19)	0.7789 (2)	0.3351 (4)	0.0180 (6)	
C4	0.1266 (2)	0.6879 (2)	0.2674 (4)	0.0201 (6)	
C4A	0.03017 (19)	0.7160 (2)	0.1659 (4)	0.0191 (6)	
C5	−0.0351 (2)	0.6377 (2)	0.0908 (4)	0.0206 (6)	
H5	−0.0180	0.5655	0.1071	0.025*	
C6	−0.1244 (2)	0.6653 (2)	−0.0068 (4)	0.0249 (7)	
H6	−0.1681	0.6119	−0.0588	0.030*	
C7	−0.1509 (2)	0.7710 (3)	−0.0296 (4)	0.0246 (7)	
H7	−0.2126	0.7889	−0.0969	0.030*	
C8	−0.0884 (2)	0.8501 (2)	0.0448 (4)	0.0232 (6)	
H8	−0.1063	0.9222	0.0311	0.028*	
C8A	0.0017 (2)	0.8203 (2)	0.1405 (4)	0.0212 (6)	
C31	0.2891 (2)	0.7728 (2)	0.4371 (4)	0.0211 (6)	
C311	0.4162 (2)	0.6460 (2)	0.5629 (4)	0.0202 (6)	
C312	0.43423 (19)	0.5536 (2)	0.6708 (4)	0.0196 (6)	
C313	0.5268 (2)	0.5248 (2)	0.7552 (4)	0.0221 (6)	
H313	0.5367	0.4627	0.8284	0.027*	
C314	0.6038 (2)	0.5870 (2)	0.7320 (4)	0.0250 (7)	
H314	0.6673	0.5677	0.7880	0.030*	
C315	0.5883 (2)	0.6778 (2)	0.6266 (4)	0.0236 (7)	
H315	0.6416	0.7203	0.6103	0.028*	
C316	0.4957 (2)	0.7078 (2)	0.5439 (4)	0.0223 (6)	
H316	0.4866	0.7711	0.4739	0.027*	

### (2b) N-(2-Nitrophenyl)-4-oxo-4H-chromene-3-carboxamide Atomic displacement parameters (Å^2^)

**Table:**

	*U*^11^	*U*^22^	*U*^33^	*U*^12^	*U*^13^	*U*^23^
O1	0.0174 (10)	0.0192 (10)	0.0275 (12)	0.0018 (8)	0.0020 (8)	0.0005 (9)
O3	0.0215 (11)	0.0186 (10)	0.0348 (13)	−0.0029 (9)	0.0027 (9)	−0.0010 (9)
O4	0.0214 (11)	0.0163 (10)	0.0381 (13)	0.0011 (9)	−0.0016 (9)	0.0017 (9)
O31	0.0355 (12)	0.0219 (11)	0.0300 (13)	0.0006 (10)	0.0022 (10)	0.0077 (10)
O32	0.0190 (10)	0.0229 (11)	0.0341 (13)	−0.0012 (9)	−0.0017 (9)	0.0027 (9)
N3	0.0160 (12)	0.0170 (12)	0.0258 (14)	0.0010 (10)	0.0004 (10)	0.0022 (10)
N31	0.0217 (12)	0.0191 (12)	0.0256 (14)	0.0015 (10)	0.0057 (11)	0.0013 (10)
C2	0.0169 (14)	0.0236 (15)	0.0223 (16)	0.0015 (12)	0.0033 (12)	−0.0012 (12)
C3	0.0173 (13)	0.0185 (13)	0.0182 (14)	−0.0004 (12)	0.0029 (11)	0.0009 (12)
C4	0.0190 (14)	0.0209 (14)	0.0210 (15)	0.0016 (12)	0.0053 (11)	0.0029 (12)
C4A	0.0162 (13)	0.0221 (14)	0.0189 (15)	−0.0015 (12)	0.0028 (11)	0.0005 (12)
C5	0.0209 (14)	0.0182 (14)	0.0229 (15)	0.0010 (12)	0.0042 (12)	0.0002 (12)
C6	0.0233 (15)	0.0272 (16)	0.0238 (16)	−0.0018 (13)	0.0033 (12)	−0.0020 (13)
C7	0.0187 (14)	0.0318 (16)	0.0236 (16)	0.0039 (14)	0.0044 (12)	0.0003 (13)
C8	0.0238 (15)	0.0252 (15)	0.0215 (15)	0.0046 (13)	0.0062 (12)	0.0007 (13)
C8A	0.0197 (14)	0.0231 (15)	0.0212 (15)	−0.0005 (12)	0.0048 (11)	−0.0019 (12)
C31	0.0204 (14)	0.0217 (14)	0.0220 (16)	−0.0012 (12)	0.0063 (12)	0.0004 (12)
C311	0.0174 (13)	0.0217 (14)	0.0207 (15)	0.0014 (12)	0.0014 (11)	−0.0012 (12)
C312	0.0177 (13)	0.0187 (14)	0.0225 (15)	−0.0012 (12)	0.0034 (11)	−0.0003 (12)
C313	0.0230 (15)	0.0211 (14)	0.0216 (16)	0.0023 (12)	0.0021 (12)	−0.0018 (12)
C314	0.0199 (14)	0.0270 (16)	0.0264 (17)	0.0041 (13)	−0.0005 (12)	−0.0063 (13)
C315	0.0175 (14)	0.0263 (15)	0.0273 (16)	−0.0025 (13)	0.0048 (12)	−0.0057 (13)
C316	0.0181 (14)	0.0217 (15)	0.0269 (17)	−0.0006 (12)	0.0033 (12)	−0.0016 (12)

### (2b) N-(2-Nitrophenyl)-4-oxo-4H-chromene-3-carboxamide Geometric parameters (Å, º)

**Table:**

O1—C2	1.346 (3)	C5—H5	0.9500
O1—C8A	1.389 (3)	C6—C7	1.395 (4)
O3—C31	1.228 (4)	C6—H6	0.9500
O4—C4	1.235 (3)	C7—C8	1.384 (4)
O31—N31	1.227 (3)	C7—H7	0.9500
O32—N31	1.244 (3)	C8—C8A	1.392 (4)
N3—C31	1.360 (4)	C8—H8	0.9500
N3—C311	1.411 (4)	C311—C316	1.396 (4)
N3—H3	0.96 (4)	C311—C312	1.413 (4)
N31—C312	1.460 (4)	C312—C313	1.392 (4)
C2—C3	1.346 (4)	C313—C314	1.378 (4)
C2—H2	0.9500	C313—H313	0.9500
C3—C4	1.468 (4)	C314—C315	1.383 (4)
C3—C31	1.508 (4)	C314—H314	0.9500
C4—C4A	1.476 (4)	C315—C316	1.394 (4)
C4A—C8A	1.385 (4)	C315—H315	0.9500
C4A—C5	1.401 (4)	C316—H316	0.9500
C5—C6	1.381 (4)		
			
C2—O1—C8A	118.3 (2)	C7—C8—C8A	117.7 (3)
C31—N3—C311	125.6 (2)	C7—C8—H8	121.2
C31—N3—H3	118 (2)	C8A—C8—H8	121.2
C311—N3—H3	116 (2)	C4A—C8A—O1	120.9 (3)
O31—N31—O32	122.1 (2)	C4A—C8A—C8	123.0 (3)
O31—N31—C312	119.0 (2)	O1—C8A—C8	116.1 (3)
O32—N31—C312	118.9 (2)	O3—C31—N3	125.1 (3)
O1—C2—C3	125.9 (3)	O3—C31—C3	120.4 (3)
O1—C2—H2	117.1	N3—C31—C3	114.5 (2)
C3—C2—H2	117.1	C316—C311—N3	121.3 (3)
C2—C3—C4	119.6 (3)	C316—C311—C312	117.0 (3)
C2—C3—C31	115.4 (3)	N3—C311—C312	121.8 (3)
C4—C3—C31	125.0 (3)	C313—C312—C311	122.0 (3)
O4—C4—C3	124.2 (3)	C313—C312—N31	115.9 (3)
O4—C4—C4A	121.8 (3)	C311—C312—N31	122.0 (2)
C3—C4—C4A	114.0 (2)	C314—C313—C312	119.5 (3)
C8A—C4A—C5	118.0 (3)	C314—C313—H313	120.2
C8A—C4A—C4	121.3 (3)	C312—C313—H313	120.2
C5—C4A—C4	120.7 (3)	C313—C314—C315	119.7 (3)
C6—C5—C4A	120.1 (3)	C313—C314—H314	120.2
C6—C5—H5	120.0	C315—C314—H314	120.2
C4A—C5—H5	120.0	C314—C315—C316	121.1 (3)
C5—C6—C7	120.5 (3)	C314—C315—H315	119.5
C5—C6—H6	119.8	C316—C315—H315	119.5
C7—C6—H6	119.8	C315—C316—C311	120.7 (3)
C8—C7—C6	120.7 (3)	C315—C316—H316	119.7
C8—C7—H7	119.6	C311—C316—H316	119.7
C6—C7—H7	119.6		
			
C8A—O1—C2—C3	−0.6 (4)	C311—N3—C31—O3	−3.2 (5)
O1—C2—C3—C4	−0.9 (5)	C311—N3—C31—C3	178.5 (3)
O1—C2—C3—C31	178.3 (3)	C2—C3—C31—O3	−1.0 (4)
C2—C3—C4—O4	−178.8 (3)	C4—C3—C31—O3	178.1 (3)
C31—C3—C4—O4	2.0 (5)	C2—C3—C31—N3	177.3 (3)
C2—C3—C4—C4A	1.3 (4)	C4—C3—C31—N3	−3.5 (4)
C31—C3—C4—C4A	−177.9 (3)	C31—N3—C311—C316	−32.2 (4)
O4—C4—C4A—C8A	179.8 (3)	C31—N3—C311—C312	149.8 (3)
C3—C4—C4A—C8A	−0.3 (4)	C316—C311—C312—C313	0.1 (4)
O4—C4—C4A—C5	−0.7 (4)	N3—C311—C312—C313	178.1 (3)
C3—C4—C4A—C5	179.2 (3)	C316—C311—C312—N31	179.4 (3)
C8A—C4A—C5—C6	0.7 (4)	N3—C311—C312—N31	−2.6 (4)
C4—C4A—C5—C6	−178.8 (3)	O31—N31—C312—C313	17.2 (4)
C4A—C5—C6—C7	−0.8 (4)	O32—N31—C312—C313	−161.3 (3)
C5—C6—C7—C8	0.1 (5)	O31—N31—C312—C311	−162.2 (3)
C6—C7—C8—C8A	0.8 (4)	O32—N31—C312—C311	19.3 (4)
C5—C4A—C8A—O1	179.3 (3)	C311—C312—C313—C314	−0.8 (4)
C4—C4A—C8A—O1	−1.2 (4)	N31—C312—C313—C314	179.8 (3)
C5—C4A—C8A—C8	0.2 (4)	C312—C313—C314—C315	0.6 (4)
C4—C4A—C8A—C8	179.7 (3)	C313—C314—C315—C316	0.3 (5)
C2—O1—C8A—C4A	1.7 (4)	C314—C315—C316—C311	−1.1 (5)
C2—O1—C8A—C8	−179.2 (3)	N3—C311—C316—C315	−177.2 (3)
C7—C8—C8A—C4A	−0.9 (5)	C312—C311—C316—C315	0.8 (4)
C7—C8—C8A—O1	179.9 (3)		

### (2b) N-(2-Nitrophenyl)-4-oxo-4H-chromene-3-carboxamide Hydrogen-bond geometry (Å, º)

**Table:**

*D*—H···*A*	*D*—H	H···*A*	*D*···*A*	*D*—H···*A*
N3—H3···O4	0.96 (4)	1.95 (4)	2.718 (3)	136 (3)
N3—H3···O32	0.96 (4)	1.96 (4)	2.633 (3)	126 (3)
C316—H316···O3	0.95	2.40	2.902 (4)	113
C8—H8···O32^i^	0.95	2.58	3.210 (4)	124
C5—H5···O1^ii^	0.95	2.60	3.375 (4)	139
C313—H313···O3^iii^	0.95	2.49	3.299 (4)	143

Symmetry codes: (i) −*x*, *y*+1/2, −*z*+1/2; (ii) −*x*, *y*−1/2, −*z*+1/2; (iii) −*x*+1, *y*−1/2, −*z*+3/2.

### (3a) N-(3-Methoxyphenyl)-4-oxo-4H-chromene-3-carboxamide Crystal data

**Table:**

C_17_H_13_NO_4_	*F*(000) = 616
*M**_r_* = 295.28	*D*_x_ = 1.487 Mg m^−^^3^
Monoclinic, *P*2_1_/*n*	Mo *K*α radiation, λ = 0.71075 Å
*a* = 9.6903 (2) Å	Cell parameters from 7535 reflections
*b* = 5.5303 (4) Å	θ = 2.4–27.5°
*c* = 24.9335 (18) Å	µ = 0.11 mm^−^^1^
β = 99.162 (5)°	*T* = 100 K
*V* = 1319.15 (14) Å^3^	Plate, yellow
*Z* = 4	0.16 × 0.11 × 0.02 mm

### (3a) N-(3-Methoxyphenyl)-4-oxo-4H-chromene-3-carboxamide Data collection

**Table:**

Rigaku Saturn724+ (2x2 bin mode) diffractometer	2665 independent reflections
Graphite Monochromator monochromator	1952 reflections with *I* > 2σ(*I*)
Detector resolution: 28.5714 pixels mm^-1^	*R*_int_ = 0.055
profile data from ω–scans	θ_max_ = 26.4°, θ_min_ = 3.0°
Absorption correction: multi-scan (*CrystalClear-SM Expert*; Rigaku, 2012)	*h* = −8→12
*T*_min_ = 0.983, *T*_max_ = 0.998	*k* = −6→4
7859 measured reflections	*l* = −31→31

### (3a) N-(3-Methoxyphenyl)-4-oxo-4H-chromene-3-carboxamide Refinement

**Table:**

Refinement on *F*^2^	0 restraints
Least-squares matrix: full	Hydrogen site location: mixed
*R*[*F*^2^ > 2σ(*F*^2^)] = 0.041	H atoms treated by a mixture of independent and constrained refinement
*wR*(*F*^2^) = 0.108	*w* = 1/[σ^2^(*F*_o_^2^) + (0.0608*P*)^2^] where *P* = (*F*_o_^2^ + 2*F*_c_^2^)/3
*S* = 0.98	(Δ/σ)_max_ < 0.001
2665 reflections	Δρ_max_ = 0.27 e Å^−^^3^
205 parameters	Δρ_min_ = −0.28 e Å^−^^3^

### (3a) N-(3-Methoxyphenyl)-4-oxo-4H-chromene-3-carboxamide Special details

**Table:**

Geometry. All e.s.d.'s (except the e.s.d. in the dihedral angle between two l.s. planes) are estimated using the full covariance matrix. The cell e.s.d.'s are taken into account individually in the estimation of e.s.d.'s in distances, angles and torsion angles; correlations between e.s.d.'s in cell parameters are only used when they are defined by crystal symmetry. An approximate (isotropic) treatment of cell e.s.d.'s is used for estimating e.s.d.'s involving l.s. planes.

### (3a) N-(3-Methoxyphenyl)-4-oxo-4H-chromene-3-carboxamide Fractional atomic coordinates and isotropic or equivalent isotropic displacement parameters (Å^2^)

**Table:**

	*x*	*y*	*z*	*U*_iso_*/*U*_eq_	
O1	0.26521 (11)	0.7928 (2)	0.57868 (4)	0.0171 (3)	
O3	0.44760 (12)	0.7936 (2)	0.44346 (5)	0.0232 (3)	
O4	0.18474 (11)	0.2131 (2)	0.47561 (5)	0.0180 (3)	
O31	0.59354 (12)	0.7141 (2)	0.26613 (5)	0.0203 (3)	
N3	0.35790 (14)	0.4246 (3)	0.41394 (6)	0.0165 (3)	
H3	0.301 (2)	0.299 (4)	0.4246 (9)	0.041 (6)*	
C2	0.32706 (16)	0.7700 (3)	0.53426 (7)	0.0161 (4)	
H2	0.3919	0.8917	0.5280	0.022 (5)*	
C3	0.30423 (16)	0.5879 (3)	0.49791 (6)	0.0142 (4)	
C4	0.20852 (16)	0.3913 (3)	0.50574 (6)	0.0142 (4)	
C4A	0.13765 (16)	0.4230 (3)	0.55341 (6)	0.0145 (4)	
C5	0.03520 (16)	0.2597 (3)	0.56506 (7)	0.0166 (4)	
H5	0.0134	0.1214	0.5427	0.020*	
C6	−0.03398 (17)	0.2980 (3)	0.60851 (7)	0.0171 (4)	
H6	−0.1032	0.1868	0.6159	0.020*	
C7	−0.00212 (17)	0.5010 (3)	0.64171 (7)	0.0187 (4)	
H7	−0.0508	0.5275	0.6714	0.022*	
C8	0.09905 (17)	0.6627 (3)	0.63182 (7)	0.0177 (4)	
H8	0.1221	0.7986	0.6548	0.021*	
C8A	0.16685 (16)	0.6224 (3)	0.58735 (7)	0.0152 (4)	
C31	0.37825 (16)	0.6126 (3)	0.44949 (6)	0.0159 (4)	
C311	0.39490 (16)	0.4120 (3)	0.36136 (6)	0.0158 (4)	
C312	0.48345 (16)	0.5805 (3)	0.34230 (7)	0.0158 (4)	
H312	0.5242	0.7080	0.3650	0.019*	
C313	0.51050 (16)	0.5572 (3)	0.28940 (7)	0.0160 (4)	
C314	0.45126 (17)	0.3717 (3)	0.25555 (7)	0.0181 (4)	
H314	0.4695	0.3599	0.2193	0.022*	
C315	0.36543 (17)	0.2046 (3)	0.27535 (7)	0.0190 (4)	
H315	0.3256	0.0763	0.2527	0.023*	
C316	0.33721 (17)	0.2231 (3)	0.32799 (7)	0.0181 (4)	
H316	0.2787	0.1074	0.3413	0.022*	
C317	0.67728 (17)	0.8800 (3)	0.30136 (7)	0.0203 (4)	
H31A	0.7369	0.9724	0.2805	0.030*	
H31B	0.7359	0.7902	0.3303	0.030*	
H31C	0.6164	0.9911	0.3174	0.030*	

### (3a) N-(3-Methoxyphenyl)-4-oxo-4H-chromene-3-carboxamide Atomic displacement parameters (Å^2^)

**Table:**

	*U*^11^	*U*^22^	*U*^33^	*U*^12^	*U*^13^	*U*^23^
O1	0.0184 (6)	0.0160 (6)	0.0181 (6)	−0.0038 (5)	0.0064 (5)	−0.0022 (5)
O3	0.0268 (7)	0.0212 (7)	0.0243 (7)	−0.0100 (6)	0.0118 (5)	−0.0044 (5)
O4	0.0198 (6)	0.0159 (6)	0.0192 (6)	−0.0040 (5)	0.0059 (5)	−0.0033 (5)
O31	0.0223 (6)	0.0216 (7)	0.0188 (6)	−0.0063 (5)	0.0087 (5)	−0.0010 (5)
N3	0.0148 (7)	0.0169 (8)	0.0192 (8)	−0.0037 (6)	0.0064 (6)	−0.0013 (6)
C2	0.0131 (8)	0.0182 (9)	0.0174 (9)	−0.0007 (7)	0.0036 (7)	0.0043 (7)
C3	0.0110 (8)	0.0155 (8)	0.0162 (8)	0.0008 (7)	0.0019 (6)	0.0021 (7)
C4	0.0121 (8)	0.0160 (9)	0.0136 (8)	0.0028 (7)	−0.0011 (6)	0.0031 (7)
C4A	0.0119 (8)	0.0149 (8)	0.0165 (8)	0.0022 (7)	0.0012 (6)	0.0022 (7)
C5	0.0144 (8)	0.0162 (9)	0.0184 (9)	−0.0013 (7)	0.0001 (7)	0.0020 (7)
C6	0.0133 (8)	0.0195 (9)	0.0185 (9)	−0.0010 (7)	0.0026 (7)	0.0056 (7)
C7	0.0172 (9)	0.0224 (10)	0.0171 (9)	0.0040 (7)	0.0049 (7)	0.0043 (7)
C8	0.0199 (9)	0.0152 (9)	0.0176 (9)	0.0020 (7)	0.0020 (7)	−0.0010 (7)
C8A	0.0119 (8)	0.0150 (9)	0.0183 (8)	−0.0009 (7)	0.0015 (6)	0.0050 (7)
C31	0.0118 (8)	0.0171 (9)	0.0185 (9)	0.0001 (7)	0.0012 (6)	0.0021 (7)
C311	0.0123 (8)	0.0175 (9)	0.0179 (9)	0.0032 (7)	0.0037 (6)	0.0009 (7)
C312	0.0123 (8)	0.0162 (9)	0.0188 (9)	−0.0007 (7)	0.0026 (6)	−0.0006 (7)
C313	0.0108 (8)	0.0166 (9)	0.0213 (9)	0.0024 (7)	0.0047 (6)	0.0023 (7)
C314	0.0172 (9)	0.0209 (9)	0.0169 (8)	0.0036 (7)	0.0049 (7)	−0.0003 (7)
C315	0.0168 (9)	0.0185 (9)	0.0214 (9)	−0.0011 (7)	0.0024 (7)	−0.0050 (7)
C316	0.0152 (8)	0.0167 (9)	0.0235 (9)	−0.0018 (7)	0.0068 (7)	−0.0004 (7)
C317	0.0174 (9)	0.0210 (10)	0.0232 (9)	−0.0051 (7)	0.0056 (7)	−0.0001 (8)

### (3a) N-(3-Methoxyphenyl)-4-oxo-4H-chromene-3-carboxamide Geometric parameters (Å, º)

**Table:**

O1—C2	1.3464 (18)	C6—H6	0.9500
O1—C8A	1.3815 (19)	C7—C8	1.378 (2)
O3—C31	1.228 (2)	C7—H7	0.9500
O4—C4	1.239 (2)	C8—C8A	1.394 (2)
O31—C313	1.3733 (19)	C8—H8	0.9500
O31—C317	1.430 (2)	C311—C316	1.396 (2)
N3—C31	1.360 (2)	C311—C312	1.400 (2)
N3—C311	1.4146 (19)	C312—C313	1.391 (2)
N3—H3	0.95 (2)	C312—H312	0.9500
C2—C3	1.349 (2)	C313—C314	1.393 (2)
C2—H2	0.9500	C314—C315	1.386 (2)
C3—C4	1.462 (2)	C314—H314	0.9500
C3—C31	1.506 (2)	C315—C316	1.386 (2)
C4—C4A	1.475 (2)	C315—H315	0.9500
C4A—C8A	1.391 (2)	C316—H316	0.9500
C4A—C5	1.406 (2)	C317—H31A	0.9800
C5—C6	1.378 (2)	C317—H31B	0.9800
C5—H5	0.9500	C317—H31C	0.9800
C6—C7	1.400 (2)		
			
C2—O1—C8A	118.25 (13)	O1—C8A—C8	116.09 (14)
C313—O31—C317	117.35 (13)	C4A—C8A—C8	122.32 (15)
C31—N3—C311	127.55 (14)	O3—C31—N3	124.66 (14)
C31—N3—H3	114.0 (13)	O3—C31—C3	120.80 (15)
C311—N3—H3	117.9 (13)	N3—C31—C3	114.51 (14)
O1—C2—C3	125.13 (15)	C316—C311—C312	120.30 (14)
O1—C2—H2	117.4	C316—C311—N3	116.92 (14)
C3—C2—H2	117.4	C312—C311—N3	122.78 (15)
C2—C3—C4	120.32 (14)	C313—C312—C311	118.59 (15)
C2—C3—C31	115.01 (15)	C313—C312—H312	120.7
C4—C3—C31	124.61 (14)	C311—C312—H312	120.7
O4—C4—C3	124.85 (14)	O31—C313—C312	123.62 (15)
O4—C4—C4A	121.19 (14)	O31—C313—C314	114.96 (14)
C3—C4—C4A	113.96 (14)	C312—C313—C314	121.40 (15)
C8A—C4A—C5	117.72 (14)	C315—C314—C313	119.22 (15)
C8A—C4A—C4	120.57 (14)	C315—C314—H314	120.4
C5—C4A—C4	121.67 (15)	C313—C314—H314	120.4
C6—C5—C4A	120.76 (16)	C314—C315—C316	120.53 (16)
C6—C5—H5	119.6	C314—C315—H315	119.7
C4A—C5—H5	119.6	C316—C315—H315	119.7
C5—C6—C7	119.94 (15)	C315—C316—C311	119.94 (15)
C5—C6—H6	120.0	C315—C316—H316	120.0
C7—C6—H6	120.0	C311—C316—H316	120.0
C8—C7—C6	120.73 (15)	O31—C317—H31A	109.5
C8—C7—H7	119.6	O31—C317—H31B	109.5
C6—C7—H7	119.6	H31A—C317—H31B	109.5
C7—C8—C8A	118.52 (15)	O31—C317—H31C	109.5
C7—C8—H8	120.7	H31A—C317—H31C	109.5
C8A—C8—H8	120.7	H31B—C317—H31C	109.5
O1—C8A—C4A	121.59 (14)		
			
C8A—O1—C2—C3	−2.3 (2)	C7—C8—C8A—O1	178.69 (14)
O1—C2—C3—C4	−1.6 (3)	C7—C8—C8A—C4A	−1.2 (3)
O1—C2—C3—C31	175.74 (14)	C311—N3—C31—O3	−8.8 (3)
C2—C3—C4—O4	−177.46 (16)	C311—N3—C31—C3	169.44 (14)
C31—C3—C4—O4	5.5 (3)	C2—C3—C31—O3	−3.5 (2)
C2—C3—C4—C4A	3.5 (2)	C4—C3—C31—O3	173.63 (15)
C31—C3—C4—C4A	−173.52 (14)	C2—C3—C31—N3	178.11 (15)
O4—C4—C4A—C8A	179.08 (15)	C4—C3—C31—N3	−4.7 (2)
C3—C4—C4A—C8A	−1.8 (2)	C31—N3—C311—C316	−166.55 (16)
O4—C4—C4A—C5	−3.4 (2)	C31—N3—C311—C312	12.6 (2)
C3—C4—C4A—C5	175.72 (14)	C316—C311—C312—C313	1.0 (2)
C8A—C4A—C5—C6	0.4 (2)	N3—C311—C312—C313	−178.12 (14)
C4—C4A—C5—C6	−177.26 (15)	C317—O31—C313—C312	12.6 (2)
C4A—C5—C6—C7	−0.2 (2)	C317—O31—C313—C314	−168.72 (14)
C5—C6—C7—C8	−0.7 (3)	C311—C312—C313—O31	178.79 (15)
C6—C7—C8—C8A	1.3 (2)	C311—C312—C313—C314	0.2 (2)
C2—O1—C8A—C4A	4.0 (2)	O31—C313—C314—C315	−179.81 (14)
C2—O1—C8A—C8	−175.85 (14)	C312—C313—C314—C315	−1.1 (2)
C5—C4A—C8A—O1	−179.53 (14)	C313—C314—C315—C316	0.8 (2)
C4—C4A—C8A—O1	−1.9 (2)	C314—C315—C316—C311	0.3 (2)
C5—C4A—C8A—C8	0.4 (2)	C312—C311—C316—C315	−1.2 (2)
C4—C4A—C8A—C8	178.01 (15)	N3—C311—C316—C315	177.90 (15)

### (3a) N-(3-Methoxyphenyl)-4-oxo-4H-chromene-3-carboxamide Hydrogen-bond geometry (Å, º)

**Table:**

*D*—H···*A*	*D*—H	H···*A*	*D*···*A*	*D*—H···*A*
N3—H3···O4	0.95 (2)	1.89 (2)	2.7147 (17)	143.8 (18)
C312—H312···O3	0.95	2.25	2.855 (2)	121
C2—H2···O3^i^	0.95	2.37	3.243 (2)	153

Symmetry code: (i) −*x*+1, −*y*+2, −*z*+1.

### (3b) N-(3-Bromophenyl)-4-oxo-4H-chromene-3-carboxamide Crystal data

**Table:**

C_16_H_10_BrNO_3_	*Z* = 4
*M**_r_* = 344.16	*F*(000) = 688
Triclinic, *P*1	*D*_x_ = 1.759 Mg m^−^^3^
*a* = 6.7435 (1) Å	Mo *K*α radiation, λ = 0.71075 Å
*b* = 7.3012 (1) Å	Cell parameters from 6848 reflections
*c* = 28.0740 (9) Å	θ = 1.5–27.5°
α = 85.309 (4)°	µ = 3.17 mm^−^^1^
β = 89.164 (4)°	*T* = 120 K
γ = 70.645 (3)°	Plate, colourless
*V* = 1299.64 (5) Å^3^	0.38 × 0.34 × 0.06 mm

### (3b) N-(3-Bromophenyl)-4-oxo-4H-chromene-3-carboxamide Data collection

**Table:**

Rigaku RAXIS conversion diffractometer	5939 independent reflections
Radiation source: Sealed Tube	5633 reflections with *I* > 2σ(*I*)
Graphite Monochromator monochromator	*R*_int_ = 0.045
Detector resolution: 10.0000 pixels mm^-1^	θ_max_ = 27.5°, θ_min_ = 1.5°
profile data from ω–scans	*h* = −7→8
Absorption correction: multi-scan (*CrystalClear-SM Expert*; Rigaku, 2012)	*k* = −9→9
*T*_min_ = 0.379, *T*_max_ = 0.833	*l* = −36→36
16781 measured reflections	

### (3b) N-(3-Bromophenyl)-4-oxo-4H-chromene-3-carboxamide Refinement

**Table:**

Refinement on *F*^2^	0 restraints
Least-squares matrix: full	Hydrogen site location: inferred from neighbouring sites
*R*[*F*^2^ > 2σ(*F*^2^)] = 0.044	H-atom parameters constrained
*wR*(*F*^2^) = 0.116	*w* = 1/[σ^2^(*F*_o_^2^) + (0.0487*P*)^2^ + 2.2824*P*] where *P* = (*F*_o_^2^ + 2*F*_c_^2^)/3
*S* = 1.08	(Δ/σ)_max_ = 0.001
5939 reflections	Δρ_max_ = 1.79 e Å^−^^3^
379 parameters	Δρ_min_ = −0.86 e Å^−^^3^

### (3b) N-(3-Bromophenyl)-4-oxo-4H-chromene-3-carboxamide Special details

**Table:**

Geometry. All e.s.d.'s (except the e.s.d. in the dihedral angle between two l.s. planes) are estimated using the full covariance matrix. The cell e.s.d.'s are taken into account individually in the estimation of e.s.d.'s in distances, angles and torsion angles; correlations between e.s.d.'s in cell parameters are only used when they are defined by crystal symmetry. An approximate (isotropic) treatment of cell e.s.d.'s is used for estimating e.s.d.'s involving l.s. planes.

### (3b) N-(3-Bromophenyl)-4-oxo-4H-chromene-3-carboxamide Fractional atomic coordinates and isotropic or equivalent isotropic displacement parameters (Å^2^)

**Table:**

	*x*	*y*	*z*	*U*_iso_*/*U*_eq_	
Br13	0.19823 (5)	0.50533 (5)	0.21591 (2)	0.02468 (10)	
O11	0.1612 (3)	0.1960 (3)	−0.09282 (8)	0.0234 (5)	
O14	0.7153 (3)	0.1950 (3)	−0.03480 (8)	0.0242 (5)	
O131	0.1525 (4)	0.3349 (4)	0.04468 (8)	0.0256 (5)	
N13	0.5020 (4)	0.3021 (4)	0.04552 (9)	0.0199 (5)	
H13	0.6134	0.2758	0.0272	0.024*	
C12	0.1726 (5)	0.2403 (4)	−0.04764 (10)	0.0213 (6)	
H12	0.0470	0.2721	−0.0297	0.026*	
C13	0.3488 (5)	0.2431 (4)	−0.02576 (10)	0.0187 (6)	
C14	0.5473 (5)	0.1936 (4)	−0.05181 (10)	0.0182 (6)	
C14A	0.5333 (5)	0.1413 (4)	−0.10070 (10)	0.0193 (6)	
C15	0.7097 (5)	0.0851 (4)	−0.12974 (11)	0.0217 (6)	
H15	0.8429	0.0789	−0.1177	0.026*	
C16	0.6925 (5)	0.0387 (5)	−0.17555 (11)	0.0258 (7)	
H16	0.8131	0.0022	−0.1951	0.031*	
C17	0.4978 (5)	0.0448 (5)	−0.19347 (11)	0.0248 (6)	
H17	0.4873	0.0114	−0.2251	0.030*	
C18	0.3209 (5)	0.0991 (4)	−0.16563 (11)	0.0227 (6)	
H18	0.1882	0.1043	−0.1778	0.027*	
C18A	0.3417 (5)	0.1459 (4)	−0.11934 (10)	0.0203 (6)	
C131	0.5316 (5)	0.3428 (4)	0.09256 (10)	0.0185 (6)	
C132	0.3685 (5)	0.4007 (4)	0.12504 (10)	0.0190 (6)	
H132	0.2278	0.4166	0.1163	0.023*	
C133	0.4191 (5)	0.4344 (4)	0.17065 (10)	0.0203 (6)	
C134	0.6201 (5)	0.4156 (4)	0.18499 (11)	0.0224 (6)	
H134	0.6488	0.4403	0.2164	0.027*	
C135	0.7799 (5)	0.3588 (5)	0.15156 (11)	0.0234 (6)	
H135	0.9200	0.3447	0.1604	0.028*	
C136	0.7379 (5)	0.3226 (4)	0.10580 (11)	0.0217 (6)	
H136	0.8483	0.2842	0.0835	0.026*	
C137	0.3224 (5)	0.2985 (4)	0.02492 (10)	0.0192 (6)	
Br23	−0.07137 (5)	1.08718 (5)	0.28460 (2)	0.02772 (10)	
O21	−0.0747 (3)	0.5655 (3)	0.59280 (7)	0.0223 (4)	
O24	0.4533 (4)	0.6676 (3)	0.54070 (8)	0.0259 (5)	
O231	−0.1079 (4)	0.8225 (4)	0.45807 (8)	0.0276 (5)	
N23	0.2310 (4)	0.8203 (4)	0.45937 (9)	0.0218 (5)	
H23	0.3452	0.7817	0.4775	0.026*	
C22	−0.0700 (5)	0.6501 (4)	0.54886 (10)	0.0211 (6)	
H22	−0.1952	0.6861	0.5301	0.025*	
C23	0.0968 (5)	0.6891 (4)	0.52871 (10)	0.0202 (6)	
C24	0.2927 (5)	0.6380 (4)	0.55608 (11)	0.0204 (6)	
C24A	0.2867 (5)	0.5466 (4)	0.60466 (10)	0.0197 (6)	
C25	0.4612 (5)	0.4903 (4)	0.63557 (11)	0.0224 (6)	
H25	0.5876	0.5111	0.6254	0.027*	
C26	0.4502 (5)	0.4048 (5)	0.68070 (11)	0.0249 (6)	
H26	0.5692	0.3671	0.7015	0.030*	
C27	0.2653 (5)	0.3732 (5)	0.69597 (11)	0.0244 (6)	
H27	0.2594	0.3149	0.7272	0.029*	
C28	0.0911 (5)	0.4256 (5)	0.66619 (11)	0.0231 (6)	
H28	−0.0345	0.4031	0.6763	0.028*	
C28A	0.1050 (5)	0.5124 (4)	0.62079 (10)	0.0206 (6)	
C231	0.2488 (5)	0.9119 (4)	0.41434 (11)	0.0213 (6)	
C232	0.0957 (5)	0.9487 (4)	0.37790 (10)	0.0212 (6)	
H232	−0.0274	0.9138	0.3826	0.025*	
C233	0.1333 (5)	1.0386 (5)	0.33470 (11)	0.0230 (6)	
C234	0.3077 (5)	1.0949 (5)	0.32614 (11)	0.0253 (6)	
H234	0.3257	1.1583	0.2963	0.030*	
C235	0.4566 (5)	1.0555 (5)	0.36291 (12)	0.0259 (7)	
H235	0.5788	1.0917	0.3580	0.031*	
C236	0.4281 (5)	0.9640 (5)	0.40655 (11)	0.0239 (6)	
H236	0.5315	0.9367	0.4312	0.029*	
C237	0.0609 (5)	0.7833 (4)	0.47874 (10)	0.0208 (6)	

### (3b) N-(3-Bromophenyl)-4-oxo-4H-chromene-3-carboxamide Atomic displacement parameters (Å^2^)

**Table:**

	*U*^11^	*U*^22^	*U*^33^	*U*^12^	*U*^13^	*U*^23^
Br13	0.02787 (18)	0.03148 (18)	0.01601 (16)	−0.01088 (13)	0.00215 (11)	−0.00539 (12)
O11	0.0200 (11)	0.0311 (12)	0.0190 (10)	−0.0076 (9)	−0.0011 (8)	−0.0052 (9)
O14	0.0211 (11)	0.0323 (12)	0.0204 (11)	−0.0096 (9)	0.0003 (8)	−0.0056 (9)
O131	0.0242 (11)	0.0364 (13)	0.0187 (10)	−0.0122 (10)	0.0021 (8)	−0.0068 (9)
N13	0.0217 (13)	0.0238 (13)	0.0145 (11)	−0.0073 (10)	0.0007 (9)	−0.0039 (9)
C12	0.0237 (15)	0.0239 (15)	0.0160 (13)	−0.0072 (12)	0.0009 (11)	−0.0038 (11)
C13	0.0200 (14)	0.0178 (13)	0.0183 (13)	−0.0062 (11)	−0.0012 (10)	−0.0016 (10)
C14	0.0221 (14)	0.0149 (13)	0.0169 (13)	−0.0054 (11)	−0.0001 (10)	−0.0002 (10)
C14A	0.0245 (15)	0.0157 (13)	0.0174 (13)	−0.0063 (11)	0.0011 (11)	−0.0006 (10)
C15	0.0210 (15)	0.0207 (14)	0.0227 (15)	−0.0061 (11)	0.0019 (11)	−0.0015 (11)
C16	0.0332 (17)	0.0206 (15)	0.0210 (15)	−0.0051 (12)	0.0065 (12)	−0.0039 (11)
C17	0.0367 (18)	0.0205 (15)	0.0155 (14)	−0.0070 (13)	−0.0001 (12)	−0.0027 (11)
C18	0.0271 (16)	0.0200 (14)	0.0190 (14)	−0.0052 (12)	−0.0045 (11)	−0.0008 (11)
C18A	0.0265 (15)	0.0173 (13)	0.0155 (13)	−0.0052 (11)	−0.0008 (11)	0.0001 (10)
C131	0.0253 (15)	0.0158 (13)	0.0148 (13)	−0.0075 (11)	−0.0018 (11)	0.0000 (10)
C132	0.0221 (14)	0.0183 (14)	0.0164 (13)	−0.0067 (11)	−0.0004 (11)	−0.0004 (10)
C133	0.0247 (15)	0.0177 (13)	0.0177 (14)	−0.0055 (11)	−0.0009 (11)	−0.0023 (10)
C134	0.0292 (16)	0.0195 (14)	0.0180 (14)	−0.0069 (12)	−0.0052 (11)	−0.0024 (11)
C135	0.0196 (15)	0.0237 (15)	0.0276 (16)	−0.0077 (12)	−0.0059 (11)	−0.0026 (12)
C136	0.0212 (15)	0.0223 (15)	0.0217 (14)	−0.0072 (11)	0.0006 (11)	−0.0016 (11)
C137	0.0226 (14)	0.0177 (14)	0.0169 (13)	−0.0059 (11)	−0.0011 (11)	−0.0016 (10)
Br23	0.02983 (19)	0.03229 (19)	0.01703 (16)	−0.00573 (13)	−0.00026 (12)	0.00149 (12)
O21	0.0225 (11)	0.0276 (11)	0.0171 (10)	−0.0087 (9)	−0.0008 (8)	−0.0010 (8)
O24	0.0219 (11)	0.0330 (13)	0.0234 (11)	−0.0108 (9)	−0.0011 (8)	0.0012 (9)
O231	0.0235 (12)	0.0375 (13)	0.0202 (11)	−0.0089 (10)	−0.0036 (9)	0.0018 (9)
N23	0.0230 (13)	0.0256 (13)	0.0151 (11)	−0.0061 (10)	−0.0028 (9)	0.0005 (10)
C22	0.0247 (15)	0.0206 (14)	0.0169 (13)	−0.0057 (11)	0.0007 (11)	−0.0039 (11)
C23	0.0229 (15)	0.0192 (14)	0.0164 (13)	−0.0038 (11)	−0.0002 (11)	−0.0029 (11)
C24	0.0218 (15)	0.0175 (13)	0.0203 (14)	−0.0039 (11)	0.0002 (11)	−0.0040 (11)
C24A	0.0220 (14)	0.0170 (13)	0.0192 (14)	−0.0046 (11)	0.0007 (11)	−0.0041 (10)
C25	0.0223 (15)	0.0206 (14)	0.0230 (15)	−0.0046 (11)	−0.0018 (11)	−0.0048 (11)
C26	0.0281 (16)	0.0216 (15)	0.0228 (15)	−0.0046 (12)	−0.0040 (12)	−0.0046 (12)
C27	0.0324 (17)	0.0221 (15)	0.0160 (14)	−0.0056 (12)	0.0003 (12)	−0.0012 (11)
C28	0.0262 (16)	0.0231 (15)	0.0196 (14)	−0.0072 (12)	0.0022 (11)	−0.0035 (11)
C28A	0.0213 (14)	0.0201 (14)	0.0188 (14)	−0.0039 (11)	−0.0013 (11)	−0.0042 (11)
C231	0.0247 (15)	0.0174 (14)	0.0191 (14)	−0.0030 (11)	0.0021 (11)	−0.0026 (11)
C232	0.0245 (15)	0.0185 (14)	0.0183 (14)	−0.0039 (11)	0.0022 (11)	−0.0018 (11)
C233	0.0219 (15)	0.0219 (15)	0.0204 (14)	−0.0007 (11)	0.0027 (11)	−0.0026 (11)
C234	0.0273 (16)	0.0222 (15)	0.0229 (15)	−0.0040 (12)	0.0069 (12)	−0.0010 (12)
C235	0.0272 (16)	0.0223 (15)	0.0281 (16)	−0.0076 (12)	0.0073 (12)	−0.0060 (12)
C236	0.0240 (15)	0.0225 (15)	0.0245 (15)	−0.0065 (12)	0.0006 (12)	−0.0039 (12)
C237	0.0247 (15)	0.0194 (14)	0.0169 (13)	−0.0050 (11)	−0.0003 (11)	−0.0030 (11)

### (3b) N-(3-Bromophenyl)-4-oxo-4H-chromene-3-carboxamide Geometric parameters (Å, º)

**Table:**

Br13—C133	1.909 (3)	Br23—C233	1.913 (3)
O11—C12	1.344 (4)	O21—C22	1.339 (4)
O11—C18A	1.376 (4)	O21—C28A	1.380 (4)
O14—C14	1.240 (4)	O24—C24	1.238 (4)
O131—C137	1.223 (4)	O231—C237	1.221 (4)
N13—C137	1.359 (4)	N23—C237	1.359 (4)
N13—C131	1.408 (4)	N23—C231	1.404 (4)
N13—H13	0.8800	N23—H23	0.8800
C12—C13	1.352 (4)	C22—C23	1.354 (4)
C12—H12	0.9500	C22—H22	0.9500
C13—C14	1.467 (4)	C23—C24	1.460 (4)
C13—C137	1.503 (4)	C23—C237	1.497 (4)
C14—C14A	1.467 (4)	C24—C24A	1.475 (4)
C14A—C18A	1.390 (4)	C24A—C28A	1.391 (4)
C14A—C15	1.397 (4)	C24A—C25	1.399 (4)
C15—C16	1.373 (4)	C25—C26	1.378 (5)
C15—H15	0.9500	C25—H25	0.9500
C16—C17	1.398 (5)	C26—C27	1.397 (5)
C16—H16	0.9500	C26—H26	0.9500
C17—C18	1.379 (5)	C27—C28	1.380 (5)
C17—H17	0.9500	C27—H27	0.9500
C18—C18A	1.391 (4)	C28—C28A	1.393 (4)
C18—H18	0.9500	C28—H28	0.9500
C131—C132	1.395 (4)	C231—C236	1.392 (5)
C131—C136	1.402 (4)	C231—C232	1.409 (4)
C132—C133	1.391 (4)	C232—C233	1.392 (4)
C132—H132	0.9500	C232—H232	0.9500
C133—C134	1.379 (5)	C233—C234	1.380 (5)
C134—C135	1.397 (5)	C234—C235	1.395 (5)
C134—H134	0.9500	C234—H234	0.9500
C135—C136	1.384 (4)	C235—C236	1.389 (5)
C135—H135	0.9500	C235—H235	0.9500
C136—H136	0.9500	C236—H236	0.9500
			
C12—O11—C18A	118.6 (2)	C22—O21—C28A	118.1 (2)
C137—N13—C131	128.0 (3)	C237—N23—C231	128.6 (3)
C137—N13—H13	116.0	C237—N23—H23	115.7
C131—N13—H13	116.0	C231—N23—H23	115.7
O11—C12—C13	125.2 (3)	O21—C22—C23	126.0 (3)
O11—C12—H12	117.4	O21—C22—H22	117.0
C13—C12—H12	117.4	C23—C22—H22	117.0
C12—C13—C14	119.6 (3)	C22—C23—C24	119.3 (3)
C12—C13—C137	115.5 (3)	C22—C23—C237	115.3 (3)
C14—C13—C137	124.9 (3)	C24—C23—C237	125.4 (3)
O14—C14—C13	123.9 (3)	O24—C24—C23	124.0 (3)
O14—C14—C14A	121.5 (3)	O24—C24—C24A	121.4 (3)
C13—C14—C14A	114.6 (3)	C23—C24—C24A	114.6 (3)
C18A—C14A—C15	118.1 (3)	C28A—C24A—C25	118.1 (3)
C18A—C14A—C14	120.3 (3)	C28A—C24A—C24	120.2 (3)
C15—C14A—C14	121.6 (3)	C25—C24A—C24	121.7 (3)
C16—C15—C14A	120.6 (3)	C26—C25—C24A	120.2 (3)
C16—C15—H15	119.7	C26—C25—H25	119.9
C14A—C15—H15	119.7	C24A—C25—H25	119.9
C15—C16—C17	120.2 (3)	C25—C26—C27	120.4 (3)
C15—C16—H16	119.9	C25—C26—H26	119.8
C17—C16—H16	119.9	C27—C26—H26	119.8
C18—C17—C16	120.6 (3)	C28—C27—C26	120.8 (3)
C18—C17—H17	119.7	C28—C27—H27	119.6
C16—C17—H17	119.7	C26—C27—H27	119.6
C17—C18—C18A	118.3 (3)	C27—C28—C28A	117.9 (3)
C17—C18—H18	120.8	C27—C28—H28	121.0
C18A—C18—H18	120.8	C28A—C28—H28	121.0
O11—C18A—C14A	121.8 (3)	O21—C28A—C24A	121.8 (3)
O11—C18A—C18	116.0 (3)	O21—C28A—C28	115.6 (3)
C14A—C18A—C18	122.2 (3)	C24A—C28A—C28	122.6 (3)
C132—C131—C136	120.4 (3)	C236—C231—N23	116.9 (3)
C132—C131—N13	123.3 (3)	C236—C231—C232	120.5 (3)
C136—C131—N13	116.3 (3)	N23—C231—C232	122.5 (3)
C133—C132—C131	117.7 (3)	C233—C232—C231	116.8 (3)
C133—C132—H132	121.2	C233—C232—H232	121.6
C131—C132—H132	121.2	C231—C232—H232	121.6
C134—C133—C132	123.5 (3)	C234—C233—C232	124.0 (3)
C134—C133—Br13	118.6 (2)	C234—C233—Br23	118.6 (2)
C132—C133—Br13	117.9 (2)	C232—C233—Br23	117.4 (2)
C133—C134—C135	117.5 (3)	C233—C234—C235	117.6 (3)
C133—C134—H134	121.3	C233—C234—H234	121.2
C135—C134—H134	121.3	C235—C234—H234	121.2
C136—C135—C134	121.2 (3)	C236—C235—C234	120.7 (3)
C136—C135—H135	119.4	C236—C235—H235	119.6
C134—C135—H135	119.4	C234—C235—H235	119.6
C135—C136—C131	119.7 (3)	C235—C236—C231	120.2 (3)
C135—C136—H136	120.2	C235—C236—H236	119.9
C131—C136—H136	120.2	C231—C236—H236	119.9
O131—C137—N13	124.8 (3)	O231—C237—N23	124.2 (3)
O131—C137—C13	121.3 (3)	O231—C237—C23	121.8 (3)
N13—C137—C13	113.8 (3)	N23—C237—C23	114.0 (3)
			
C18A—O11—C12—C13	1.2 (5)	C28A—O21—C22—C23	0.2 (4)
O11—C12—C13—C14	−0.5 (5)	O21—C22—C23—C24	−0.4 (5)
O11—C12—C13—C137	179.6 (3)	O21—C22—C23—C237	179.5 (3)
C12—C13—C14—O14	179.0 (3)	C22—C23—C24—O24	179.9 (3)
C137—C13—C14—O14	−1.2 (5)	C237—C23—C24—O24	0.0 (5)
C12—C13—C14—C14A	−0.9 (4)	C22—C23—C24—C24A	−0.2 (4)
C137—C13—C14—C14A	179.0 (3)	C237—C23—C24—C24A	179.9 (3)
O14—C14—C14A—C18A	−178.3 (3)	O24—C24—C24A—C28A	−179.1 (3)
C13—C14—C14A—C18A	1.6 (4)	C23—C24—C24A—C28A	1.1 (4)
O14—C14—C14A—C15	2.0 (5)	O24—C24—C24A—C25	0.3 (4)
C13—C14—C14A—C15	−178.2 (3)	C23—C24—C24A—C25	−179.5 (3)
C18A—C14A—C15—C16	0.9 (5)	C28A—C24A—C25—C26	−0.4 (4)
C14—C14A—C15—C16	−179.3 (3)	C24—C24A—C25—C26	−179.8 (3)
C14A—C15—C16—C17	−0.8 (5)	C24A—C25—C26—C27	0.1 (5)
C15—C16—C17—C18	0.6 (5)	C25—C26—C27—C28	0.4 (5)
C16—C17—C18—C18A	−0.4 (5)	C26—C27—C28—C28A	−0.7 (5)
C12—O11—C18A—C14A	−0.4 (4)	C22—O21—C28A—C24A	0.7 (4)
C12—O11—C18A—C18	179.2 (3)	C22—O21—C28A—C28	179.8 (3)
C15—C14A—C18A—O11	178.8 (3)	C25—C24A—C28A—O21	179.2 (3)
C14—C14A—C18A—O11	−1.0 (4)	C24—C24A—C28A—O21	−1.4 (4)
C15—C14A—C18A—C18	−0.8 (4)	C25—C24A—C28A—C28	0.2 (4)
C14—C14A—C18A—C18	179.4 (3)	C24—C24A—C28A—C28	179.6 (3)
C17—C18—C18A—O11	−179.0 (3)	C27—C28—C28A—O21	−178.7 (3)
C17—C18—C18A—C14A	0.6 (5)	C27—C28—C28A—C24A	0.3 (5)
C137—N13—C131—C132	−4.7 (5)	C237—N23—C231—C236	168.5 (3)
C137—N13—C131—C136	175.4 (3)	C237—N23—C231—C232	−12.4 (5)
C136—C131—C132—C133	−0.8 (4)	C236—C231—C232—C233	−0.2 (4)
N13—C131—C132—C133	179.3 (3)	N23—C231—C232—C233	−179.3 (3)
C131—C132—C133—C134	0.7 (5)	C231—C232—C233—C234	−1.0 (4)
C131—C132—C133—Br13	−178.0 (2)	C231—C232—C233—Br23	179.4 (2)
C132—C133—C134—C135	−0.3 (5)	C232—C233—C234—C235	1.3 (5)
Br13—C133—C134—C135	178.4 (2)	Br23—C233—C234—C235	−179.0 (2)
C133—C134—C135—C136	−0.1 (5)	C233—C234—C235—C236	−0.4 (5)
C134—C135—C136—C131	−0.1 (5)	C234—C235—C236—C231	−0.7 (5)
C132—C131—C136—C135	0.5 (5)	N23—C231—C236—C235	−179.9 (3)
N13—C131—C136—C135	−179.6 (3)	C232—C231—C236—C235	1.0 (4)
C131—N13—C137—O131	1.5 (5)	C231—N23—C237—O231	2.0 (5)
C131—N13—C137—C13	−178.2 (3)	C231—N23—C237—C23	−177.8 (3)
C12—C13—C137—O131	1.1 (4)	C22—C23—C237—O231	0.4 (4)
C14—C13—C137—O131	−178.8 (3)	C24—C23—C237—O231	−179.8 (3)
C12—C13—C137—N13	−179.2 (3)	C22—C23—C237—N23	−179.8 (3)
C14—C13—C137—N13	0.9 (4)	C24—C23—C237—N23	0.0 (4)

### (3b) N-(3-Bromophenyl)-4-oxo-4H-chromene-3-carboxamide Hydrogen-bond geometry (Å, º)

**Table:**

*D*—H···*A*	*D*—H	H···*A*	*D*···*A*	*D*—H···*A*
N13—H13···O14	0.88	1.93	2.686 (3)	143
N23—H23···O24	0.88	1.94	2.698 (3)	143
C12—H12···O131	0.95	2.34	2.727 (4)	104
C22—H22···O231	0.95	2.33	2.725 (4)	104
C132—H132···O131	0.95	2.26	2.860 (4)	121
C232—H232···O231	0.95	2.28	2.865 (4)	119
C12—H12···O14^i^	0.95	2.49	3.221 (4)	134
C22—H22···O24^i^	0.95	2.43	3.185 (4)	136
C15—H15···O11^ii^	0.95	2.68	3.587 (4)	160
C25—H25···O21^ii^	0.95	2.58	3.530 (4)	177
C136—H136···O131^ii^	0.95	2.43	3.282 (4)	149
C236—H236···O231^ii^	0.95	2.41	3.270 (4)	151

Symmetry codes: (i) *x*−1, *y*, *z*; (ii) *x*+1, *y*, *z*.

### (4a) N-(4-Methoxyphenyl)-4-oxo-4H-chromene-3-carboxamide Crystal data

**Table:**

C_17_H_13_NO_4_	*F*(000) = 616
*M**_r_* = 295.28	*D*_x_ = 1.506 Mg m^−^^3^
Monoclinic, *P*2_1_/*n*	Mo *K*α radiation, λ = 0.71075 Å
*a* = 14.1629 (10) Å	Cell parameters from 15826 reflections
*b* = 6.772 (5) Å	θ = 2.6–27.5°
*c* = 15.1898 (11) Å	µ = 0.11 mm^−^^1^
β = 116.607 (11)°	*T* = 100 K
*V* = 1302.6 (10) Å^3^	Plate, colourless
*Z* = 4	0.15 × 0.07 × 0.01 mm

### (4a) N-(4-Methoxyphenyl)-4-oxo-4H-chromene-3-carboxamide Data collection

**Table:**

Rigaku Saturn724+ (2x2 bin mode) diffractometer	2987 independent reflections
Graphite Monochromator monochromator	2617 reflections with *I* > 2σ(*I*)
Detector resolution: 28.5714 pixels mm^-1^	*R*_int_ = 0.042
profile data from ω–scans	θ_max_ = 27.5°, θ_min_ = 2.7°
Absorption correction: multi-scan (*CrystalClear-SM Expert*; Rigaku, 2012)	*h* = −18→16
*T*_min_ = 0.984, *T*_max_ = 0.999	*k* = −8→8
16554 measured reflections	*l* = −19→19

### (4a) N-(4-Methoxyphenyl)-4-oxo-4H-chromene-3-carboxamide Refinement

**Table:**

Refinement on *F*^2^	0 restraints
Least-squares matrix: full	Hydrogen site location: mixed
*R*[*F*^2^ > 2σ(*F*^2^)] = 0.037	H atoms treated by a mixture of independent and constrained refinement
*wR*(*F*^2^) = 0.103	*w* = 1/[σ^2^(*F*_o_^2^) + (0.0587*P*)^2^ + 0.664*P*] where *P* = (*F*_o_^2^ + 2*F*_c_^2^)/3
*S* = 0.92	(Δ/σ)_max_ = 0.005
2987 reflections	Δρ_max_ = 0.39 e Å^−^^3^
204 parameters	Δρ_min_ = −0.18 e Å^−^^3^

### (4a) N-(4-Methoxyphenyl)-4-oxo-4H-chromene-3-carboxamide Special details

**Table:**

Geometry. All e.s.d.'s (except the e.s.d. in the dihedral angle between two l.s. planes) are estimated using the full covariance matrix. The cell e.s.d.'s are taken into account individually in the estimation of e.s.d.'s in distances, angles and torsion angles; correlations between e.s.d.'s in cell parameters are only used when they are defined by crystal symmetry. An approximate (isotropic) treatment of cell e.s.d.'s is used for estimating e.s.d.'s involving l.s. planes.

### (4a) N-(4-Methoxyphenyl)-4-oxo-4H-chromene-3-carboxamide Fractional atomic coordinates and isotropic or equivalent isotropic displacement parameters (Å^2^)

**Table:**

	*x*	*y*	*z*	*U*_iso_*/*U*_eq_	
O1	0.61512 (6)	0.04824 (12)	0.04192 (6)	0.0222 (2)	
O4	0.63888 (6)	0.60878 (12)	0.15264 (6)	0.0221 (2)	
O314	0.64113 (7)	0.68423 (13)	0.66556 (6)	0.0260 (2)	
O3	0.61112 (8)	0.10549 (13)	0.30685 (7)	0.0281 (2)	
N3	0.63425 (8)	0.44104 (15)	0.31133 (7)	0.0206 (2)	
H3	0.6347 (12)	0.541 (3)	0.2725 (12)	0.034 (4)*	
C2	0.62017 (9)	0.08077 (18)	0.13108 (8)	0.0207 (2)	
H2	0.6193	−0.0316	0.1681	0.025*	
C3	0.62644 (8)	0.26035 (17)	0.17274 (8)	0.0196 (2)	
C4	0.63203 (8)	0.43834 (17)	0.12051 (8)	0.0187 (2)	
C4A	0.62683 (8)	0.40077 (17)	0.02284 (8)	0.0192 (2)	
C5	0.62976 (9)	0.55604 (18)	−0.03704 (9)	0.0219 (2)	
H5	0.6382	0.6881	−0.0136	0.026*	
C6	0.62051 (9)	0.51790 (19)	−0.12981 (9)	0.0239 (3)	
H6	0.6233	0.6235	−0.1697	0.029*	
C7	0.60702 (9)	0.32347 (19)	−0.16527 (9)	0.0241 (3)	
H7	0.5993	0.2987	−0.2297	0.029*	
C8	0.60487 (9)	0.16788 (18)	−0.10756 (9)	0.0233 (3)	
H8	0.5961	0.0361	−0.1314	0.028*	
C8A	0.61592 (8)	0.20886 (17)	−0.01329 (8)	0.0199 (2)	
C31	0.62381 (9)	0.25962 (17)	0.27073 (9)	0.0206 (2)	
C311	0.63093 (8)	0.49421 (17)	0.40033 (8)	0.0194 (2)	
C312	0.64385 (9)	0.36073 (18)	0.47503 (8)	0.0218 (2)	
H312	0.6515	0.2237	0.4665	0.026*	
C313	0.64547 (9)	0.42952 (18)	0.56178 (9)	0.0221 (2)	
H313	0.6542	0.3385	0.6125	0.027*	
C314	0.63439 (9)	0.63048 (18)	0.57565 (8)	0.0207 (2)	
C315	0.61990 (9)	0.76285 (17)	0.50058 (8)	0.0215 (2)	
H315	0.6110	0.8996	0.5086	0.026*	
C316	0.61854 (9)	0.69445 (17)	0.41378 (8)	0.0208 (2)	
H316	0.6090	0.7854	0.3629	0.025*	
C317	0.62986 (11)	0.88959 (19)	0.67957 (9)	0.0273 (3)	
H31A	0.6389	0.9118	0.7466	0.041*	
H31B	0.5594	0.9338	0.6321	0.041*	
H31C	0.6835	0.9643	0.6696	0.041*	

### (4a) N-(4-Methoxyphenyl)-4-oxo-4H-chromene-3-carboxamide Atomic displacement parameters (Å^2^)

**Table:**

	*U*^11^	*U*^22^	*U*^33^	*U*^12^	*U*^13^	*U*^23^
O1	0.0260 (4)	0.0173 (4)	0.0241 (4)	−0.0006 (3)	0.0119 (3)	−0.0015 (3)
O4	0.0272 (4)	0.0173 (4)	0.0224 (4)	−0.0011 (3)	0.0116 (3)	−0.0010 (3)
O314	0.0354 (5)	0.0222 (4)	0.0223 (4)	0.0018 (4)	0.0148 (4)	0.0009 (3)
O3	0.0406 (5)	0.0187 (4)	0.0294 (5)	−0.0028 (4)	0.0195 (4)	0.0016 (3)
N3	0.0246 (5)	0.0176 (5)	0.0194 (5)	−0.0011 (4)	0.0097 (4)	0.0011 (4)
C2	0.0205 (5)	0.0191 (5)	0.0222 (5)	0.0002 (4)	0.0092 (4)	0.0010 (4)
C3	0.0186 (5)	0.0182 (5)	0.0208 (5)	0.0003 (4)	0.0077 (4)	0.0007 (4)
C4	0.0158 (5)	0.0180 (5)	0.0205 (5)	0.0001 (4)	0.0065 (4)	−0.0002 (4)
C4A	0.0166 (5)	0.0198 (5)	0.0206 (5)	0.0007 (4)	0.0077 (4)	0.0000 (4)
C5	0.0211 (5)	0.0204 (5)	0.0242 (5)	0.0006 (4)	0.0100 (4)	0.0008 (4)
C6	0.0234 (5)	0.0251 (6)	0.0240 (6)	0.0016 (5)	0.0114 (5)	0.0033 (5)
C7	0.0224 (5)	0.0301 (7)	0.0209 (5)	0.0009 (5)	0.0106 (4)	−0.0019 (5)
C8	0.0210 (5)	0.0241 (6)	0.0245 (6)	−0.0003 (4)	0.0100 (4)	−0.0039 (5)
C8A	0.0170 (5)	0.0196 (5)	0.0227 (5)	0.0003 (4)	0.0084 (4)	0.0002 (4)
C31	0.0192 (5)	0.0194 (5)	0.0222 (5)	0.0001 (4)	0.0084 (4)	0.0008 (4)
C311	0.0184 (5)	0.0203 (5)	0.0187 (5)	−0.0014 (4)	0.0077 (4)	−0.0004 (4)
C312	0.0223 (5)	0.0187 (5)	0.0231 (6)	−0.0004 (4)	0.0089 (4)	0.0017 (4)
C313	0.0236 (6)	0.0207 (6)	0.0218 (5)	−0.0001 (4)	0.0100 (4)	0.0041 (4)
C314	0.0195 (5)	0.0230 (6)	0.0193 (5)	−0.0011 (4)	0.0085 (4)	−0.0001 (4)
C315	0.0219 (5)	0.0189 (5)	0.0226 (5)	0.0008 (4)	0.0088 (4)	0.0002 (4)
C316	0.0217 (5)	0.0189 (5)	0.0201 (5)	−0.0005 (4)	0.0077 (4)	0.0033 (4)
C317	0.0366 (7)	0.0231 (6)	0.0255 (6)	0.0020 (5)	0.0166 (5)	−0.0013 (5)

### (4a) N-(4-Methoxyphenyl)-4-oxo-4H-chromene-3-carboxamide Geometric parameters (Å, º)

**Table:**

O1—C2	1.3420 (14)	C6—H6	0.9500
O1—C8A	1.3767 (15)	C7—C8	1.3797 (19)
O4—C4	1.2399 (16)	C7—H7	0.9500
O314—C314	1.3747 (14)	C8—C8A	1.3969 (16)
O314—C317	1.4266 (18)	C8—H8	0.9500
O3—C31	1.2293 (16)	C311—C316	1.3941 (19)
N3—C31	1.3528 (17)	C311—C312	1.3971 (16)
N3—C311	1.4201 (15)	C312—C313	1.3881 (17)
N3—H3	0.901 (17)	C312—H312	0.9500
C2—C3	1.3553 (18)	C313—C314	1.3967 (19)
C2—H2	0.9500	C313—H313	0.9500
C3—C4	1.4645 (17)	C314—C315	1.3916 (17)
C3—C31	1.5055 (16)	C315—C316	1.3894 (16)
C4—C4A	1.4742 (16)	C315—H315	0.9500
C4A—C8A	1.3921 (18)	C316—H316	0.9500
C4A—C5	1.4031 (17)	C317—H31A	0.9800
C5—C6	1.3799 (17)	C317—H31B	0.9800
C5—H5	0.9500	C317—H31C	0.9800
C6—C7	1.403 (2)		
			
C2—O1—C8A	118.29 (10)	O1—C8A—C8	116.09 (11)
C314—O314—C317	116.34 (9)	C4A—C8A—C8	122.10 (11)
C31—N3—C311	128.37 (10)	O3—C31—N3	125.15 (11)
C31—N3—H3	114.6 (10)	O3—C31—C3	121.16 (11)
C311—N3—H3	116.4 (10)	N3—C31—C3	113.68 (10)
O1—C2—C3	125.53 (11)	C316—C311—C312	119.31 (11)
O1—C2—H2	117.2	C316—C311—N3	116.50 (10)
C3—C2—H2	117.2	C312—C311—N3	124.13 (11)
C2—C3—C4	119.58 (11)	C313—C312—C311	119.62 (12)
C2—C3—C31	115.67 (10)	C313—C312—H312	120.2
C4—C3—C31	124.72 (10)	C311—C312—H312	120.2
O4—C4—C3	124.51 (11)	C312—C313—C314	121.00 (11)
O4—C4—C4A	121.10 (10)	C312—C313—H313	119.5
C3—C4—C4A	114.37 (10)	C314—C313—H313	119.5
C8A—C4A—C5	118.31 (11)	O314—C314—C315	124.24 (12)
C8A—C4A—C4	120.31 (10)	O314—C314—C313	116.45 (10)
C5—C4A—C4	121.36 (11)	C315—C314—C313	119.30 (11)
C6—C5—C4A	120.33 (11)	C316—C315—C314	119.81 (12)
C6—C5—H5	119.8	C316—C315—H315	120.1
C4A—C5—H5	119.8	C314—C315—H315	120.1
C5—C6—C7	120.14 (11)	C315—C316—C311	120.94 (11)
C5—C6—H6	119.9	C315—C316—H316	119.5
C7—C6—H6	119.9	C311—C316—H316	119.5
C8—C7—C6	120.71 (12)	O314—C317—H31A	109.5
C8—C7—H7	119.6	O314—C317—H31B	109.5
C6—C7—H7	119.6	H31A—C317—H31B	109.5
C7—C8—C8A	118.38 (12)	O314—C317—H31C	109.5
C7—C8—H8	120.8	H31A—C317—H31C	109.5
C8A—C8—H8	120.8	H31B—C317—H31C	109.5
O1—C8A—C4A	121.81 (11)		
			
C8A—O1—C2—C3	−0.22 (17)	C7—C8—C8A—O1	−178.88 (10)
O1—C2—C3—C4	−2.25 (18)	C7—C8—C8A—C4A	1.28 (17)
O1—C2—C3—C31	176.01 (10)	C311—N3—C31—O3	−1.40 (19)
C2—C3—C4—O4	−179.33 (11)	C311—N3—C31—C3	177.21 (10)
C31—C3—C4—O4	2.58 (18)	C2—C3—C31—O3	−3.68 (17)
C2—C3—C4—C4A	1.79 (15)	C4—C3—C31—O3	174.48 (11)
C31—C3—C4—C4A	−176.31 (10)	C2—C3—C31—N3	177.65 (10)
O4—C4—C4A—C8A	−178.03 (10)	C4—C3—C31—N3	−4.19 (16)
C3—C4—C4A—C8A	0.89 (15)	C31—N3—C311—C316	−165.10 (11)
O4—C4—C4A—C5	0.21 (17)	C31—N3—C311—C312	17.83 (18)
C3—C4—C4A—C5	179.13 (10)	C316—C311—C312—C313	−0.79 (17)
C8A—C4A—C5—C6	0.89 (17)	N3—C311—C312—C313	176.21 (10)
C4—C4A—C5—C6	−177.38 (10)	C311—C312—C313—C314	−0.02 (17)
C4A—C5—C6—C7	0.64 (18)	C317—O314—C314—C315	1.46 (16)
C5—C6—C7—C8	−1.26 (18)	C317—O314—C314—C313	179.96 (11)
C6—C7—C8—C8A	0.31 (17)	C312—C313—C314—O314	−177.58 (10)
C2—O1—C8A—C4A	3.11 (15)	C312—C313—C314—C315	1.00 (17)
C2—O1—C8A—C8	−176.72 (9)	O314—C314—C315—C316	177.30 (10)
C5—C4A—C8A—O1	178.30 (10)	C313—C314—C315—C316	−1.16 (17)
C4—C4A—C8A—O1	−3.41 (16)	C314—C315—C316—C311	0.36 (17)
C5—C4A—C8A—C8	−1.88 (16)	C312—C311—C316—C315	0.63 (17)
C4—C4A—C8A—C8	176.42 (10)	N3—C311—C316—C315	−176.60 (10)

### (4a) N-(4-Methoxyphenyl)-4-oxo-4H-chromene-3-carboxamide Hydrogen-bond geometry (Å, º)

**Table:**

*D*—H···*A*	*D*—H	H···*A*	*D*···*A*	*D*—H···*A*
N3—H3···O4	0.901 (17)	1.903 (16)	2.6919 (13)	145.0 (15)
C312—H312···O3	0.95	2.37	2.9441 (17)	119
C2—H2···O4^i^	0.95	2.47	3.212 (3)	134
C316—H316···O3^ii^	0.95	2.33	3.201 (2)	152

Symmetry codes: (i) *x*, *y*−1, *z*; (ii) *x*, *y*+1, *z*.

### (4d) N-(4-Methylphenyl)-4-oxo-4H-chromene-3-carboxamide Crystal data

**Table:**

C_17_H_13_NO_3_	*Z* = 2
*M**_r_* = 279.28	*F*(000) = 292
Triclinic, *P*1	*D*_x_ = 1.418 Mg m^−^^3^
*a* = 6.6106 (5) Å	Mo *K*α radiation, λ = 0.71075 Å
*b* = 7.0143 (5) Å	Cell parameters from 8940 reflections
*c* = 15.3749 (11) Å	θ = 3.2–27.5°
α = 91.444 (6)°	µ = 0.10 mm^−^^1^
β = 95.238 (6)°	*T* = 100 K
γ = 112.551 (8)°	Plate, colourless
*V* = 654.25 (9) Å^3^	0.16 × 0.09 × 0.02 mm

### (4d) N-(4-Methylphenyl)-4-oxo-4H-chromene-3-carboxamide Data collection

**Table:**

Rigaku Saturn724+ (2x2 bin mode) diffractometer	2986 independent reflections
Radiation source: Sealed Tube	2645 reflections with *I* > 2σ(*I*)
Mirrors monochromator	*R*_int_ = 0.035
Detector resolution: 28.5714 pixels mm^-1^	θ_max_ = 27.6°, θ_min_ = 3.2°
profile data from ω–scans	*h* = −8→8
Absorption correction: multi-scan (*CrystalClear-SM Expert*; Rigaku, 2012)	*k* = −9→8
*T*_min_ = 0.985, *T*_max_ = 0.998	*l* = −19→19
9400 measured reflections	

### (4d) N-(4-Methylphenyl)-4-oxo-4H-chromene-3-carboxamide Refinement

**Table:**

Refinement on *F*^2^	0 restraints
Least-squares matrix: full	Hydrogen site location: mixed
*R*[*F*^2^ > 2σ(*F*^2^)] = 0.043	H atoms treated by a mixture of independent and constrained refinement
*wR*(*F*^2^) = 0.123	*w* = 1/[σ^2^(*F*_o_^2^) + (0.0687*P*)^2^ + 0.1454*P*] where *P* = (*F*_o_^2^ + 2*F*_c_^2^)/3
*S* = 1.08	(Δ/σ)_max_ = 0.004
2986 reflections	Δρ_max_ = 0.33 e Å^−^^3^
196 parameters	Δρ_min_ = −0.26 e Å^−^^3^

### (4d) N-(4-Methylphenyl)-4-oxo-4H-chromene-3-carboxamide Special details

**Table:**

Geometry. All e.s.d.'s (except the e.s.d. in the dihedral angle between two l.s. planes) are estimated using the full covariance matrix. The cell e.s.d.'s are taken into account individually in the estimation of e.s.d.'s in distances, angles and torsion angles; correlations between e.s.d.'s in cell parameters are only used when they are defined by crystal symmetry. An approximate (isotropic) treatment of cell e.s.d.'s is used for estimating e.s.d.'s involving l.s. planes.

### (4d) N-(4-Methylphenyl)-4-oxo-4H-chromene-3-carboxamide Fractional atomic coordinates and isotropic or equivalent isotropic displacement parameters (Å^2^)

**Table:**

	*x*	*y*	*z*	*U*_iso_*/*U*_eq_	
O1	0.82225 (13)	0.28691 (13)	0.53040 (5)	0.0234 (2)	
O3	0.75229 (13)	0.36263 (14)	0.27108 (6)	0.0285 (2)	
O4	0.21316 (13)	0.23497 (13)	0.42176 (5)	0.0245 (2)	
N3	0.38310 (16)	0.28969 (15)	0.26610 (6)	0.0217 (2)	
H3	0.282 (3)	0.276 (3)	0.3036 (11)	0.043 (5)*	
C2	0.78092 (18)	0.30515 (17)	0.44464 (8)	0.0220 (2)	
H2	0.8985	0.3286	0.4097	0.032 (4)*	
C3	0.58640 (17)	0.29301 (16)	0.40379 (7)	0.0197 (2)	
C4	0.39777 (18)	0.24920 (16)	0.45362 (7)	0.0198 (2)	
C4A	0.44234 (18)	0.22256 (16)	0.54690 (7)	0.0197 (2)	
C5	0.27553 (19)	0.17259 (18)	0.60293 (8)	0.0230 (2)	
H5	0.1298	0.1528	0.5803	0.028*	
C6	0.3225 (2)	0.15213 (19)	0.69049 (8)	0.0264 (3)	
H6	0.2086	0.1160	0.7279	0.032*	
C7	0.5381 (2)	0.18446 (19)	0.72459 (8)	0.0274 (3)	
H7	0.5697	0.1734	0.7853	0.033*	
C8	0.7048 (2)	0.23214 (18)	0.67093 (8)	0.0256 (3)	
H8	0.8509	0.2541	0.6939	0.031*	
C8A	0.65314 (18)	0.24727 (17)	0.58227 (8)	0.0210 (2)	
C311	0.32431 (18)	0.28817 (17)	0.17506 (7)	0.0222 (2)	
C312	0.4436 (2)	0.24849 (19)	0.11163 (8)	0.0271 (3)	
H312	0.5759	0.2289	0.1284	0.032*	
C313	0.3675 (2)	0.23785 (19)	0.02375 (8)	0.0296 (3)	
H313	0.4502	0.2118	−0.0191	0.036*	
C314	0.1735 (2)	0.26422 (18)	−0.00357 (8)	0.0273 (3)	
C315	0.0570 (2)	0.30296 (19)	0.06084 (8)	0.0263 (3)	
H315	−0.0762	0.3208	0.0440	0.032*	
C316	0.13057 (19)	0.31625 (18)	0.14920 (8)	0.0241 (3)	
H316	0.0489	0.3445	0.1919	0.029*	
C317	0.0915 (2)	0.2486 (2)	−0.09925 (8)	0.0335 (3)	
H31C	−0.0432	0.2766	−0.1057	0.050*	
H31D	0.0598	0.1090	−0.1244	0.050*	
H31E	0.2045	0.3500	−0.1299	0.050*	
C31	0.58315 (18)	0.31961 (17)	0.30716 (8)	0.0216 (2)	

### (4d) N-(4-Methylphenyl)-4-oxo-4H-chromene-3-carboxamide Atomic displacement parameters (Å^2^)

**Table:**

	*U*^11^	*U*^22^	*U*^33^	*U*^12^	*U*^13^	*U*^23^
O1	0.0174 (4)	0.0264 (4)	0.0264 (4)	0.0091 (3)	0.0004 (3)	0.0004 (3)
O3	0.0202 (4)	0.0359 (5)	0.0297 (5)	0.0098 (4)	0.0080 (3)	0.0047 (4)
O4	0.0174 (4)	0.0332 (5)	0.0252 (4)	0.0120 (3)	0.0028 (3)	0.0048 (3)
N3	0.0194 (5)	0.0256 (5)	0.0213 (5)	0.0096 (4)	0.0042 (4)	0.0033 (4)
C2	0.0188 (5)	0.0203 (5)	0.0261 (6)	0.0068 (4)	0.0027 (4)	0.0001 (4)
C3	0.0182 (5)	0.0166 (5)	0.0245 (6)	0.0069 (4)	0.0033 (4)	0.0010 (4)
C4	0.0178 (5)	0.0172 (5)	0.0249 (5)	0.0073 (4)	0.0021 (4)	0.0013 (4)
C4A	0.0201 (5)	0.0168 (5)	0.0226 (5)	0.0080 (4)	0.0011 (4)	0.0001 (4)
C5	0.0222 (5)	0.0221 (5)	0.0264 (6)	0.0104 (4)	0.0031 (4)	0.0011 (4)
C6	0.0296 (6)	0.0267 (6)	0.0254 (6)	0.0129 (5)	0.0060 (5)	0.0014 (4)
C7	0.0343 (6)	0.0268 (6)	0.0221 (5)	0.0140 (5)	−0.0007 (5)	−0.0006 (4)
C8	0.0251 (6)	0.0250 (6)	0.0267 (6)	0.0114 (5)	−0.0042 (4)	−0.0026 (4)
C8A	0.0202 (5)	0.0175 (5)	0.0257 (6)	0.0078 (4)	0.0020 (4)	−0.0004 (4)
C311	0.0224 (5)	0.0203 (5)	0.0218 (5)	0.0058 (4)	0.0036 (4)	0.0020 (4)
C312	0.0245 (6)	0.0278 (6)	0.0285 (6)	0.0091 (5)	0.0059 (4)	0.0011 (5)
C313	0.0313 (6)	0.0297 (6)	0.0253 (6)	0.0077 (5)	0.0102 (5)	−0.0003 (5)
C314	0.0318 (6)	0.0217 (5)	0.0228 (6)	0.0040 (5)	0.0037 (5)	0.0019 (4)
C315	0.0269 (6)	0.0256 (6)	0.0253 (6)	0.0093 (5)	0.0012 (4)	0.0025 (4)
C316	0.0245 (6)	0.0252 (6)	0.0233 (6)	0.0099 (5)	0.0044 (4)	0.0020 (4)
C317	0.0425 (7)	0.0301 (6)	0.0224 (6)	0.0083 (6)	0.0029 (5)	0.0009 (5)
C31	0.0201 (5)	0.0198 (5)	0.0257 (6)	0.0078 (4)	0.0047 (4)	0.0026 (4)

### (4d) N-(4-Methylphenyl)-4-oxo-4H-chromene-3-carboxamide Geometric parameters (Å, º)

**Table:**

O1—C2	1.3414 (14)	C7—C8	1.3799 (18)
O1—C8A	1.3779 (14)	C7—H7	0.9500
O3—C31	1.2296 (14)	C8—C8A	1.3914 (16)
O4—C4	1.2386 (13)	C8—H8	0.9500
N3—C31	1.3488 (14)	C311—C316	1.3935 (16)
N3—C311	1.4168 (14)	C311—C312	1.3948 (16)
N3—H3	0.900 (18)	C312—C313	1.3878 (17)
C2—C3	1.3494 (15)	C312—H312	0.9500
C2—H2	0.9500	C313—C314	1.3939 (18)
C3—C4	1.4590 (15)	C313—H313	0.9500
C3—C31	1.5013 (16)	C314—C315	1.3907 (17)
C4—C4A	1.4688 (15)	C314—C317	1.5068 (16)
C4A—C8A	1.3926 (15)	C315—C316	1.3889 (16)
C4A—C5	1.4047 (16)	C315—H315	0.9500
C5—C6	1.3773 (16)	C316—H316	0.9500
C5—H5	0.9500	C317—H31C	0.9800
C6—C7	1.4023 (17)	C317—H31D	0.9800
C6—H6	0.9500	C317—H31E	0.9800
			
C2—O1—C8A	118.52 (9)	O1—C8A—C4A	121.24 (10)
C31—N3—C311	127.52 (10)	C8—C8A—C4A	122.38 (11)
C31—N3—H3	112.7 (11)	C316—C311—C312	119.49 (11)
C311—N3—H3	119.7 (11)	C316—C311—N3	117.29 (10)
O1—C2—C3	125.51 (10)	C312—C311—N3	123.11 (10)
O1—C2—H2	117.2	C313—C312—C311	119.44 (11)
C3—C2—H2	117.2	C313—C312—H312	120.3
C2—C3—C4	119.57 (10)	C311—C312—H312	120.3
C2—C3—C31	115.13 (10)	C312—C313—C314	122.04 (11)
C4—C3—C31	125.27 (10)	C312—C313—H313	119.0
O4—C4—C3	124.11 (10)	C314—C313—H313	119.0
O4—C4—C4A	121.31 (10)	C315—C314—C313	117.49 (11)
C3—C4—C4A	114.58 (9)	C315—C314—C317	121.24 (12)
C8A—C4A—C5	118.12 (10)	C313—C314—C317	121.26 (12)
C8A—C4A—C4	120.48 (10)	C316—C315—C314	121.62 (11)
C5—C4A—C4	121.39 (10)	C316—C315—H315	119.2
C6—C5—C4A	120.25 (11)	C314—C315—H315	119.2
C6—C5—H5	119.9	C315—C316—C311	119.91 (11)
C4A—C5—H5	119.9	C315—C316—H316	120.0
C5—C6—C7	120.20 (11)	C311—C316—H316	120.0
C5—C6—H6	119.9	C314—C317—H31C	109.5
C7—C6—H6	119.9	C314—C317—H31D	109.5
C8—C7—C6	120.76 (11)	H31C—C317—H31D	109.5
C8—C7—H7	119.6	C314—C317—H31E	109.5
C6—C7—H7	119.6	H31C—C317—H31E	109.5
C7—C8—C8A	118.23 (11)	H31D—C317—H31E	109.5
C7—C8—H8	120.9	O3—C31—N3	124.93 (11)
C8A—C8—H8	120.9	O3—C31—C3	120.73 (10)
O1—C8A—C8	116.38 (10)	N3—C31—C3	114.33 (9)
			
C8A—O1—C2—C3	1.72 (17)	C4—C4A—C8A—O1	−3.26 (16)
O1—C2—C3—C4	−2.37 (17)	C5—C4A—C8A—C8	−2.55 (17)
O1—C2—C3—C31	179.75 (9)	C4—C4A—C8A—C8	177.21 (10)
C2—C3—C4—O4	−179.91 (10)	C31—N3—C311—C316	−161.26 (11)
C31—C3—C4—O4	−2.27 (18)	C31—N3—C311—C312	22.52 (18)
C2—C3—C4—C4A	0.18 (15)	C316—C311—C312—C313	0.07 (18)
C31—C3—C4—C4A	177.83 (9)	N3—C311—C312—C313	176.21 (10)
O4—C4—C4A—C8A	−177.42 (10)	C311—C312—C313—C314	−0.46 (19)
C3—C4—C4A—C8A	2.48 (15)	C312—C313—C314—C315	0.26 (19)
O4—C4—C4A—C5	2.32 (17)	C312—C313—C314—C317	−178.96 (11)
C3—C4—C4A—C5	−177.77 (10)	C313—C314—C315—C316	0.35 (18)
C8A—C4A—C5—C6	0.93 (17)	C317—C314—C315—C316	179.57 (11)
C4—C4A—C5—C6	−178.83 (10)	C314—C315—C316—C311	−0.73 (18)
C4A—C5—C6—C7	1.07 (18)	C312—C311—C316—C315	0.51 (18)
C5—C6—C7—C8	−1.57 (18)	N3—C311—C316—C315	−175.85 (10)
C6—C7—C8—C8A	0.02 (18)	C311—N3—C31—O3	4.35 (19)
C2—O1—C8A—C8	−179.25 (9)	C311—N3—C31—C3	−174.78 (10)
C2—O1—C8A—C4A	1.19 (15)	C2—C3—C31—O3	−4.51 (16)
C7—C8—C8A—O1	−177.48 (10)	C4—C3—C31—O3	177.76 (10)
C7—C8—C8A—C4A	2.07 (17)	C2—C3—C31—N3	174.67 (10)
C5—C4A—C8A—O1	176.98 (10)	C4—C3—C31—N3	−3.07 (16)

### (4d) N-(4-Methylphenyl)-4-oxo-4H-chromene-3-carboxamide Hydrogen-bond geometry (Å, º)

**Table:**

*D*—H···*A*	*D*—H	H···*A*	*D*···*A*	*D*—H···*A*
N3—H3···O4	0.900 (18)	1.916 (18)	2.7098 (13)	146.1 (15)
C312—H312···O3	0.95	2.37	2.9240 (16)	116
C2—H2···O4^i^	0.95	2.40	3.1280 (14)	133
C316—H316···O3^ii^	0.95	2.44	3.3644 (14)	164

Symmetry codes: (i) *x*+1, *y*, *z*; (ii) *x*−1, *y*, *z*.

### (4e) N-(4-Hydroxyphenyl)-4-oxo-4H-chromene-3-carboxamide Crystal data

**Table:**

C_16_H_11_NO_4_	*Z* = 4
*M**_r_* = 281.26	*F*(000) = 584
Triclinic, *P*1	*D*_x_ = 1.494 Mg m^−^^3^
*a* = 7.0756 (5) Å	Mo *K*α radiation, λ = 0.71075 Å
*b* = 12.5125 (9) Å	Cell parameters from 14545 reflections
*c* = 14.2944 (10) Å	θ = 2.9–27.5°
α = 86.267 (8)°	µ = 0.11 mm^−^^1^
β = 83.839 (8)°	*T* = 100 K
γ = 84.588 (8)°	Block, colourless
*V* = 1250.68 (16) Å^3^	0.14 × 0.04 × 0.04 mm

### (4e) N-(4-Hydroxyphenyl)-4-oxo-4H-chromene-3-carboxamide Data collection

**Table:**

Rigaku Saturn724+ (2x2 bin mode) diffractometer	5627 measured reflections
Radiation source: Sealed Tube	5627 independent reflections
Mirrors monochromator	4343 reflections with *I* > 2σ(*I*)
Detector resolution: 28.5714 pixels mm^-1^	θ_max_ = 27.6°, θ_min_ = 2.9°
profile data from ω–scans	*h* = −9→9
Absorption correction: multi-scan (*CrystalClear-SM Expert*; Rigaku, 2012)	*k* = −16→16
*T*_min_ = 0.985, *T*_max_ = 0.996	*l* = −4→18

### (4e) N-(4-Hydroxyphenyl)-4-oxo-4H-chromene-3-carboxamide Refinement

**Table:**

Refinement on *F*^2^	0 restraints
Least-squares matrix: full	Hydrogen site location: mixed
*R*[*F*^2^ > 2σ(*F*^2^)] = 0.085	H atoms treated by a mixture of independent and constrained refinement
*wR*(*F*^2^) = 0.252	*w* = 1/[σ^2^(*F*_o_^2^) + (0.1127*P*)^2^ + 0.9725*P*] where *P* = (*F*_o_^2^ + 2*F*_c_^2^)/3
*S* = 1.18	(Δ/σ)_max_ < 0.001
5627 reflections	Δρ_max_ = 0.41 e Å^−^^3^
392 parameters	Δρ_min_ = −0.38 e Å^−^^3^

### (4e) N-(4-Hydroxyphenyl)-4-oxo-4H-chromene-3-carboxamide Special details

**Table:**

Geometry. All e.s.d.'s (except the e.s.d. in the dihedral angle between two l.s. planes) are estimated using the full covariance matrix. The cell e.s.d.'s are taken into account individually in the estimation of e.s.d.'s in distances, angles and torsion angles; correlations between e.s.d.'s in cell parameters are only used when they are defined by crystal symmetry. An approximate (isotropic) treatment of cell e.s.d.'s is used for estimating e.s.d.'s involving l.s. planes.
Refinement. Refined as a 2-component twin. 2-axis (0 0 1) [-1 0 5], Angle () [] = 3.22 Deg, Freq = 48 ************* (-1.000 0.000 0.000) (h1) (h2) Nr Overlap = 1085 (0.000 - 1.000 0.000) * (k1) = (k2) BASF = 0.40 (-0.412 - 0.127 1.000) (l1) (l2) DEL-*R* =-0.068

### (4e) N-(4-Hydroxyphenyl)-4-oxo-4H-chromene-3-carboxamide Fractional atomic coordinates and isotropic or equivalent isotropic displacement parameters (Å^2^)

**Table:**

	*x*	*y*	*z*	*U*_iso_*/*U*_eq_	
O11	0.8738 (4)	−0.0689 (2)	−0.21765 (17)	0.0295 (6)	
O13	0.7750 (4)	−0.0154 (2)	0.06141 (17)	0.0339 (6)	
O14	0.5168 (4)	0.1945 (2)	−0.14104 (17)	0.0311 (6)	
O114	0.4248 (4)	0.2817 (2)	0.40213 (18)	0.0379 (7)	
H114	0.353 (8)	0.344 (5)	0.412 (4)	0.057*	
N13	0.5991 (4)	0.1441 (2)	0.0370 (2)	0.0270 (6)	
H13	0.546 (6)	0.188 (3)	−0.011 (3)	0.032*	
C12	0.8371 (5)	−0.0430 (3)	−0.1277 (2)	0.0280 (7)	
H12	0.8983	−0.0883	−0.0820	0.034*	
C13	0.7215 (5)	0.0413 (3)	−0.0963 (2)	0.0254 (7)	
C14	0.6239 (5)	0.1147 (3)	−0.1638 (2)	0.0258 (7)	
C14A	0.6635 (5)	0.0847 (3)	−0.2619 (2)	0.0259 (7)	
C15	0.5814 (5)	0.1460 (3)	−0.3350 (3)	0.0311 (8)	
H15	0.4998	0.2090	−0.3212	0.037*	
C16	0.6177 (6)	0.1161 (3)	−0.4271 (3)	0.0342 (8)	
H16	0.5602	0.1578	−0.4761	0.041*	
C17	0.7399 (6)	0.0235 (3)	−0.4478 (3)	0.0360 (8)	
H17	0.7644	0.0031	−0.5111	0.043*	
C18	0.8250 (5)	−0.0382 (3)	−0.3772 (3)	0.0330 (8)	
H18	0.9083	−0.1005	−0.3910	0.040*	
C18A	0.7842 (5)	−0.0058 (3)	−0.2852 (2)	0.0274 (7)	
C111	0.5570 (5)	0.1758 (3)	0.1308 (2)	0.0252 (7)	
C112	0.6421 (5)	0.1236 (3)	0.2077 (2)	0.0282 (7)	
H112	0.7315	0.0626	0.1985	0.034*	
C113	0.5955 (5)	0.1611 (3)	0.2966 (2)	0.0294 (7)	
H113	0.6544	0.1259	0.3482	0.035*	
C114	0.4642 (5)	0.2494 (3)	0.3121 (2)	0.0288 (7)	
C115	0.3799 (5)	0.3017 (3)	0.2363 (3)	0.0295 (8)	
H115	0.2910	0.3628	0.2458	0.035*	
C116	0.4259 (5)	0.2644 (3)	0.1463 (2)	0.0288 (7)	
H116	0.3669	0.2999	0.0948	0.035*	
C131	0.7013 (5)	0.0543 (3)	0.0076 (2)	0.0256 (7)	
O21	0.3577 (4)	0.4196 (2)	0.68202 (17)	0.0291 (6)	
O23	0.2438 (4)	0.4764 (2)	0.40951 (18)	0.0340 (6)	
O24	0.0699 (4)	0.7076 (2)	0.61185 (18)	0.0329 (6)	
O214	−0.0597 (4)	0.7840 (2)	0.07027 (17)	0.0335 (6)	
H214	−0.127 (7)	0.846 (4)	0.061 (4)	0.050*	
N23	0.1222 (4)	0.6485 (2)	0.4321 (2)	0.0271 (6)	
H23	0.083 (6)	0.697 (4)	0.476 (3)	0.033*	
C22	0.3224 (5)	0.4471 (3)	0.5933 (2)	0.0279 (7)	
H22	0.3692	0.3977	0.5467	0.033*	
C23	0.2255 (5)	0.5396 (3)	0.5643 (2)	0.0251 (7)	
C24	0.1521 (5)	0.6191 (3)	0.6325 (2)	0.0269 (7)	
C24A	0.1811 (5)	0.5846 (3)	0.7308 (2)	0.0265 (7)	
C25	0.1072 (5)	0.6482 (3)	0.8058 (2)	0.0292 (7)	
H25	0.0377	0.7154	0.7934	0.035*	
C26	0.1346 (6)	0.6140 (3)	0.8973 (3)	0.0339 (8)	
H26	0.0833	0.6574	0.9476	0.041*	
C27	0.2386 (6)	0.5147 (3)	0.9163 (3)	0.0358 (9)	
H27	0.2567	0.4913	0.9796	0.043*	
C28	0.3146 (6)	0.4509 (3)	0.8432 (3)	0.0310 (8)	
H28	0.3866	0.3844	0.8552	0.037*	
C28A	0.2824 (5)	0.4874 (3)	0.7521 (2)	0.0269 (7)	
C211	0.0775 (5)	0.6804 (3)	0.3389 (2)	0.0267 (7)	
C212	0.1618 (5)	0.6283 (3)	0.2592 (2)	0.0282 (7)	
H212	0.2525	0.5680	0.2658	0.034*	
C213	0.1131 (5)	0.6645 (3)	0.1709 (3)	0.0310 (8)	
H213	0.1699	0.6284	0.1170	0.037*	
C214	−0.0177 (5)	0.7531 (3)	0.1599 (2)	0.0273 (7)	
C215	−0.0999 (5)	0.8059 (3)	0.2387 (2)	0.0281 (7)	
H215	−0.1887	0.8670	0.2317	0.034*	
C216	−0.0524 (5)	0.7694 (3)	0.3276 (2)	0.0279 (7)	
H216	−0.1093	0.8057	0.3813	0.033*	
C231	0.1984 (5)	0.5522 (3)	0.4620 (2)	0.0257 (7)	

### (4e) N-(4-Hydroxyphenyl)-4-oxo-4H-chromene-3-carboxamide Atomic displacement parameters (Å^2^)

**Table:**

	*U*^11^	*U*^22^	*U*^33^	*U*^12^	*U*^13^	*U*^23^
O11	0.0371 (14)	0.0262 (13)	0.0243 (12)	0.0048 (10)	−0.0052 (10)	−0.0025 (10)
O13	0.0470 (16)	0.0281 (14)	0.0255 (12)	0.0065 (11)	−0.0081 (11)	−0.0009 (10)
O14	0.0372 (14)	0.0265 (13)	0.0289 (12)	0.0033 (11)	−0.0047 (10)	−0.0017 (10)
O114	0.0536 (17)	0.0314 (15)	0.0277 (13)	0.0109 (12)	−0.0098 (12)	−0.0072 (11)
N13	0.0329 (15)	0.0240 (15)	0.0234 (13)	0.0013 (12)	−0.0042 (11)	0.0009 (11)
C12	0.0331 (18)	0.0278 (18)	0.0233 (16)	−0.0015 (14)	−0.0053 (13)	−0.0008 (13)
C13	0.0286 (16)	0.0230 (16)	0.0253 (16)	−0.0045 (13)	−0.0052 (13)	0.0004 (13)
C14	0.0253 (16)	0.0244 (17)	0.0273 (16)	−0.0035 (13)	−0.0028 (13)	0.0024 (13)
C14A	0.0292 (17)	0.0231 (17)	0.0254 (16)	−0.0047 (13)	−0.0022 (13)	−0.0001 (13)
C15	0.0351 (19)	0.0292 (19)	0.0286 (17)	−0.0024 (15)	−0.0032 (14)	0.0005 (14)
C16	0.041 (2)	0.035 (2)	0.0261 (17)	−0.0012 (16)	−0.0059 (15)	0.0024 (15)
C17	0.045 (2)	0.039 (2)	0.0234 (16)	−0.0030 (17)	−0.0032 (15)	−0.0015 (15)
C18	0.037 (2)	0.033 (2)	0.0286 (17)	−0.0003 (15)	−0.0015 (15)	−0.0049 (15)
C18A	0.0289 (17)	0.0305 (18)	0.0231 (15)	−0.0050 (14)	−0.0048 (13)	0.0033 (13)
C111	0.0290 (17)	0.0232 (16)	0.0240 (15)	−0.0033 (13)	−0.0029 (13)	−0.0034 (13)
C112	0.0320 (18)	0.0245 (17)	0.0281 (16)	0.0012 (14)	−0.0050 (13)	−0.0032 (13)
C113	0.0398 (19)	0.0228 (17)	0.0259 (16)	0.0016 (14)	−0.0096 (14)	0.0006 (13)
C114	0.0355 (18)	0.0255 (17)	0.0258 (16)	−0.0010 (14)	−0.0038 (14)	−0.0053 (13)
C115	0.0342 (18)	0.0230 (17)	0.0313 (18)	0.0040 (14)	−0.0077 (14)	−0.0039 (14)
C116	0.0353 (18)	0.0248 (17)	0.0266 (16)	0.0008 (14)	−0.0072 (14)	−0.0022 (13)
C131	0.0300 (17)	0.0216 (16)	0.0253 (16)	−0.0020 (13)	−0.0045 (13)	−0.0003 (13)
O21	0.0383 (14)	0.0233 (12)	0.0252 (12)	0.0029 (10)	−0.0067 (10)	−0.0012 (10)
O23	0.0462 (15)	0.0276 (13)	0.0275 (12)	0.0057 (11)	−0.0067 (11)	−0.0044 (10)
O24	0.0414 (15)	0.0269 (13)	0.0299 (13)	0.0050 (11)	−0.0084 (11)	−0.0028 (10)
O214	0.0446 (15)	0.0296 (14)	0.0250 (12)	0.0064 (11)	−0.0081 (11)	0.0007 (10)
N23	0.0357 (16)	0.0221 (15)	0.0238 (14)	0.0006 (12)	−0.0068 (12)	−0.0009 (11)
C22	0.0329 (18)	0.0267 (18)	0.0249 (16)	−0.0022 (14)	−0.0055 (13)	−0.0031 (14)
C23	0.0281 (16)	0.0226 (16)	0.0248 (15)	−0.0009 (13)	−0.0047 (13)	−0.0030 (13)
C24	0.0298 (17)	0.0245 (17)	0.0270 (16)	−0.0016 (13)	−0.0065 (13)	−0.0005 (13)
C24A	0.0296 (17)	0.0243 (17)	0.0261 (16)	−0.0035 (13)	−0.0035 (13)	−0.0015 (13)
C25	0.0345 (18)	0.0258 (18)	0.0276 (17)	−0.0008 (14)	−0.0055 (14)	−0.0027 (14)
C26	0.043 (2)	0.0299 (19)	0.0290 (18)	−0.0007 (16)	−0.0042 (15)	−0.0051 (15)
C27	0.050 (2)	0.034 (2)	0.0243 (17)	−0.0014 (17)	−0.0085 (16)	0.0007 (15)
C28	0.041 (2)	0.0238 (17)	0.0287 (17)	−0.0023 (15)	−0.0056 (15)	0.0010 (14)
C28A	0.0318 (17)	0.0247 (17)	0.0247 (16)	−0.0036 (14)	−0.0038 (13)	−0.0027 (13)
C211	0.0302 (17)	0.0250 (17)	0.0253 (16)	−0.0033 (13)	−0.0052 (13)	0.0005 (13)
C212	0.0314 (17)	0.0239 (17)	0.0285 (17)	0.0038 (13)	−0.0046 (14)	−0.0017 (14)
C213	0.0369 (19)	0.0283 (18)	0.0270 (17)	0.0025 (15)	−0.0021 (14)	−0.0042 (14)
C214	0.0312 (17)	0.0262 (17)	0.0246 (16)	−0.0014 (14)	−0.0066 (13)	0.0018 (13)
C215	0.0305 (17)	0.0229 (17)	0.0306 (17)	0.0006 (13)	−0.0054 (14)	−0.0005 (13)
C216	0.0328 (18)	0.0247 (17)	0.0263 (16)	−0.0014 (14)	−0.0041 (13)	−0.0026 (13)
C231	0.0292 (17)	0.0243 (17)	0.0236 (15)	−0.0003 (13)	−0.0046 (12)	−0.0022 (13)

### (4e) N-(4-Hydroxyphenyl)-4-oxo-4H-chromene-3-carboxamide Geometric parameters (Å, º)

**Table:**

O11—C12	1.339 (4)	O21—C22	1.336 (4)
O11—C18A	1.377 (4)	O21—C28A	1.384 (4)
O13—C131	1.241 (4)	O23—C231	1.244 (4)
O14—C14	1.235 (4)	O24—C24	1.234 (4)
O114—C114	1.366 (4)	O214—C214	1.369 (4)
O114—H114	0.91 (6)	O214—H214	0.88 (5)
N13—C131	1.343 (4)	N23—C231	1.337 (4)
N13—C111	1.416 (4)	N23—C211	1.424 (4)
N13—H13	0.94 (4)	N23—H23	0.90 (4)
C12—C13	1.343 (5)	C22—C23	1.353 (5)
C12—H12	0.9500	C22—H22	0.9500
C13—C14	1.469 (5)	C23—C24	1.459 (5)
C13—C131	1.495 (5)	C23—C231	1.492 (5)
C14—C14A	1.466 (5)	C24—C24A	1.473 (5)
C14A—C18A	1.389 (5)	C24A—C28A	1.386 (5)
C14A—C15	1.401 (5)	C24A—C25	1.403 (5)
C15—C16	1.383 (5)	C25—C26	1.377 (5)
C15—H15	0.9500	C25—H25	0.9500
C16—C17	1.406 (6)	C26—C27	1.409 (5)
C16—H16	0.9500	C26—H26	0.9500
C17—C18	1.385 (5)	C27—C28	1.388 (5)
C17—H17	0.9500	C27—H27	0.9500
C18—C18A	1.394 (5)	C28—C28A	1.387 (5)
C18—H18	0.9500	C28—H28	0.9500
C111—C116	1.390 (5)	C211—C216	1.388 (5)
C111—C112	1.405 (5)	C211—C212	1.399 (5)
C112—C113	1.379 (5)	C212—C213	1.381 (5)
C112—H112	0.9500	C212—H212	0.9500
C113—C114	1.388 (5)	C213—C214	1.387 (5)
C113—H113	0.9500	C213—H213	0.9500
C114—C115	1.389 (5)	C214—C215	1.388 (5)
C115—C116	1.392 (5)	C215—C216	1.388 (5)
C115—H115	0.9500	C215—H215	0.9500
C116—H116	0.9500	C216—H216	0.9500
			
C12—O11—C18A	118.3 (3)	C22—O21—C28A	118.5 (3)
C114—O114—H114	119 (3)	C214—O214—H214	118 (3)
C131—N13—C111	127.5 (3)	C231—N23—C211	126.8 (3)
C131—N13—H13	114 (3)	C231—N23—H23	118 (3)
C111—N13—H13	118 (3)	C211—N23—H23	115 (3)
O11—C12—C13	125.8 (3)	O21—C22—C23	125.3 (3)
O11—C12—H12	117.1	O21—C22—H22	117.3
C13—C12—H12	117.1	C23—C22—H22	117.3
C12—C13—C14	119.6 (3)	C22—C23—C24	119.8 (3)
C12—C13—C131	116.6 (3)	C22—C23—C231	116.2 (3)
C14—C13—C131	123.8 (3)	C24—C23—C231	124.0 (3)
O14—C14—C14A	122.2 (3)	O24—C24—C23	124.3 (3)
O14—C14—C13	123.7 (3)	O24—C24—C24A	121.5 (3)
C14A—C14—C13	114.0 (3)	C23—C24—C24A	114.2 (3)
C18A—C14A—C15	117.8 (3)	C28A—C24A—C25	117.8 (3)
C18A—C14A—C14	120.9 (3)	C28A—C24A—C24	120.7 (3)
C15—C14A—C14	121.3 (3)	C25—C24A—C24	121.6 (3)
C16—C15—C14A	120.8 (4)	C26—C25—C24A	120.6 (3)
C16—C15—H15	119.6	C26—C25—H25	119.7
C14A—C15—H15	119.6	C24A—C25—H25	119.7
C15—C16—C17	119.7 (3)	C25—C26—C27	120.1 (3)
C15—C16—H16	120.2	C25—C26—H26	120.0
C17—C16—H16	120.2	C27—C26—H26	120.0
C18—C17—C16	121.0 (3)	C28—C27—C26	120.3 (3)
C18—C17—H17	119.5	C28—C27—H27	119.8
C16—C17—H17	119.5	C26—C27—H27	119.8
C17—C18—C18A	117.7 (4)	C28A—C28—C27	118.0 (3)
C17—C18—H18	121.1	C28A—C28—H28	121.0
C18A—C18—H18	121.1	C27—C28—H28	121.0
O11—C18A—C14A	121.3 (3)	O21—C28A—C24A	121.2 (3)
O11—C18A—C18	115.6 (3)	O21—C28A—C28	115.6 (3)
C14A—C18A—C18	123.1 (3)	C24A—C28A—C28	123.2 (3)
C116—C111—C112	118.9 (3)	C216—C211—C212	119.1 (3)
C116—C111—N13	117.5 (3)	C216—C211—N23	117.7 (3)
C112—C111—N13	123.5 (3)	C212—C211—N23	123.2 (3)
C113—C112—C111	119.7 (3)	C213—C212—C211	120.0 (3)
C113—C112—H112	120.1	C213—C212—H212	120.0
C111—C112—H112	120.1	C211—C212—H212	120.0
C112—C113—C114	121.3 (3)	C212—C213—C214	120.8 (3)
C112—C113—H113	119.3	C212—C213—H213	119.6
C114—C113—H113	119.3	C214—C213—H213	119.6
O114—C114—C113	117.9 (3)	O214—C214—C213	117.5 (3)
O114—C114—C115	122.8 (3)	O214—C214—C215	123.1 (3)
C113—C114—C115	119.3 (3)	C213—C214—C215	119.4 (3)
C114—C115—C116	119.8 (3)	C216—C215—C214	120.0 (3)
C114—C115—H115	120.1	C216—C215—H215	120.0
C116—C115—H115	120.1	C214—C215—H215	120.0
C111—C116—C115	120.9 (3)	C215—C216—C211	120.7 (3)
C111—C116—H116	119.5	C215—C216—H216	119.7
C115—C116—H116	119.5	C211—C216—H216	119.7
O13—C131—N13	123.6 (3)	O23—C231—N23	123.3 (3)
O13—C131—C13	120.5 (3)	O23—C231—C23	121.0 (3)
N13—C131—C13	115.9 (3)	N23—C231—C23	115.6 (3)
			
C18A—O11—C12—C13	−1.2 (5)	C28A—O21—C22—C23	3.5 (5)
O11—C12—C13—C14	−0.4 (6)	O21—C22—C23—C24	0.6 (6)
O11—C12—C13—C131	179.8 (3)	O21—C22—C23—C231	−178.4 (3)
C12—C13—C14—O14	−179.3 (3)	C22—C23—C24—O24	176.5 (4)
C131—C13—C14—O14	0.5 (5)	C231—C23—C24—O24	−4.6 (6)
C12—C13—C14—C14A	1.1 (5)	C22—C23—C24—C24A	−4.4 (5)
C131—C13—C14—C14A	−179.2 (3)	C231—C23—C24—C24A	174.6 (3)
O14—C14—C14A—C18A	−179.8 (3)	O24—C24—C24A—C28A	−176.6 (3)
C13—C14—C14A—C18A	−0.2 (5)	C23—C24—C24A—C28A	4.3 (5)
O14—C14—C14A—C15	0.2 (5)	O24—C24—C24A—C25	3.7 (5)
C13—C14—C14A—C15	179.9 (3)	C23—C24—C24A—C25	−175.4 (3)
C18A—C14A—C15—C16	1.0 (5)	C28A—C24A—C25—C26	−0.3 (5)
C14—C14A—C15—C16	−179.1 (3)	C24—C24A—C25—C26	179.4 (3)
C14A—C15—C16—C17	−0.6 (6)	C24A—C25—C26—C27	0.4 (6)
C15—C16—C17—C18	−0.1 (6)	C25—C26—C27—C28	0.2 (6)
C16—C17—C18—C18A	0.4 (6)	C26—C27—C28—C28A	−0.9 (6)
C12—O11—C18A—C14A	2.1 (5)	C22—O21—C28A—C24A	−3.5 (5)
C12—O11—C18A—C18	−178.7 (3)	C22—O21—C28A—C28	176.2 (3)
C15—C14A—C18A—O11	178.6 (3)	C25—C24A—C28A—O21	179.2 (3)
C14—C14A—C18A—O11	−1.4 (5)	C24—C24A—C28A—O21	−0.5 (5)
C15—C14A—C18A—C18	−0.6 (5)	C25—C24A—C28A—C28	−0.5 (5)
C14—C14A—C18A—C18	179.4 (3)	C24—C24A—C28A—C28	179.8 (3)
C17—C18—C18A—O11	−179.3 (3)	C27—C28—C28A—O21	−178.6 (3)
C17—C18—C18A—C14A	−0.1 (6)	C27—C28—C28A—C24A	1.1 (6)
C131—N13—C111—C116	−170.9 (3)	C231—N23—C211—C216	160.2 (3)
C131—N13—C111—C112	9.9 (6)	C231—N23—C211—C212	−21.6 (6)
C116—C111—C112—C113	−0.4 (5)	C216—C211—C212—C213	−1.0 (5)
N13—C111—C112—C113	178.8 (3)	N23—C211—C212—C213	−179.2 (3)
C111—C112—C113—C114	0.6 (6)	C211—C212—C213—C214	0.6 (6)
C112—C113—C114—O114	179.8 (3)	C212—C213—C214—O214	−179.6 (3)
C112—C113—C114—C115	−0.8 (6)	C212—C213—C214—C215	0.2 (6)
O114—C114—C115—C116	−179.8 (4)	O214—C214—C215—C216	179.2 (3)
C113—C114—C115—C116	0.8 (6)	C213—C214—C215—C216	−0.6 (5)
C112—C111—C116—C115	0.5 (5)	C214—C215—C216—C211	0.2 (5)
N13—C111—C116—C115	−178.8 (3)	C212—C211—C216—C215	0.6 (5)
C114—C115—C116—C111	−0.7 (6)	N23—C211—C216—C215	178.9 (3)
C111—N13—C131—O13	0.1 (6)	C211—N23—C231—O23	1.2 (6)
C111—N13—C131—C13	179.5 (3)	C211—N23—C231—C23	−178.4 (3)
C12—C13—C131—O13	−6.3 (5)	C22—C23—C231—O23	7.8 (5)
C14—C13—C131—O13	173.9 (3)	C24—C23—C231—O23	−171.1 (3)
C12—C13—C131—N13	174.3 (3)	C22—C23—C231—N23	−172.6 (3)
C14—C13—C131—N13	−5.5 (5)	C24—C23—C231—N23	8.5 (5)

### (4e) N-(4-Hydroxyphenyl)-4-oxo-4H-chromene-3-carboxamide Hydrogen-bond geometry (Å, º)

**Table:**

*D*—H···*A*	*D*—H	H···*A*	*D*···*A*	*D*—H···*A*
N13—H13···O14	0.94 (4)	1.88 (4)	2.693 (4)	143 (4)
N23—H23···O24	0.90 (4)	1.95 (4)	2.698 (4)	139 (4)
C112—H112···O13	0.95	2.23	2.833 (4)	121
C212—H212···O23	0.95	2.28	2.845 (4)	117
O114—H114···O23	0.91 (6)	1.76 (6)	2.647 (4)	167 (5)
O214—H214···O13^i^	0.88 (5)	1.81 (5)	2.668 (4)	165 (5)
C16—H16···O114^ii^	0.95	2.46	3.411 (5)	174
C18—H18···O24^iii^	0.95	2.56	3.481 (5)	163
C22—H22···O114	0.95	2.58	3.508 (4)	166
C26—H26···O214^iv^	0.95	2.51	3.454 (5)	175
C28—H28···O14^iv^	0.95	2.46	3.391 (5)	165

Symmetry codes: (i) *x*−1, *y*+1, *z*; (ii) *x*, *y*, *z*−1; (iii) *x*+1, *y*−1, *z*−1; (iv) *x*, *y*, *z*+1.
